# Using Intervention Mapping to Develop an mHealth Intervention to Support Men Who Have Sex With Men Engaging in Chemsex (Budd): Development and Usability Study

**DOI:** 10.2196/39678

**Published:** 2022-12-21

**Authors:** Corinne Herrijgers, Tom Platteau, Heidi Vandebosch, Karolien Poels, Eric Florence

**Affiliations:** 1 Department of Clinical Sciences Institute of Tropical Medicine Antwerpen Belgium; 2 Department of Communication Studies University of Antwerp Antwerpen Belgium

**Keywords:** mobile health, chemsex, intervention mapping, harm reduction, men who have sex with men, intervention, mobile phone

## Abstract

**Background:**

Chemsex refers to the intentional use of drugs before or during sex among men who have sex with men (MSM). Engaging in chemsex has been linked to significant negative impacts on physical, psychological, and social well-being. However, no evidence-based support tools have addressed either these harms or the care needs of MSM who engage in chemsex.

**Objective:**

The purpose of this paper was to describe the development of a mobile health intervention (named *Budd*) using the intervention mapping protocol (IMP). Budd aims to support and inform MSM who participate in chemsex, reduce the negative impacts associated with chemsex, and encourage more reasoned participation.

**Methods:**

The IMP consists of 6 steps to develop, implement, and evaluate evidence-based health interventions. A needs assessment was carried out between September 2, 2019, and March 31, 2020, by conducting a literature study and in-depth interviews. Change objectives were selected based on these findings, after which theory-based intervention methods were selected. The first version of the intervention was developed in December 2020 and pilot-tested between February 1, 2021, and April 30, 2021. Adjustments were made based on the findings from this study. A separate article will be dedicated to the effectiveness study, conducted between October 15, 2021, and February 24, 2022, and implementation of the intervention. The Budd app went live in April 2022.

**Results:**

Budd aims to address individual factors and support chemsex participants in applying harm reduction measures when taking drugs (drug information, drug combination tool, and notebook), preparing for participation in a chemsex session (articles on chemsex, preparation tool, and event-specific checklist), planning sufficient time after a chemsex session to recover (planning tool), seeking support for their chemsex participation (overview of existing local health care and peer support services, reflection, personal statistics, and user testimonials), taking HIV medication or pre-exposure prophylaxis in a timely manner during a chemsex session (preparation tool), and contacting emergency services in case of an emergency and giving first aid to others (emergency information and personal buddy).

**Conclusions:**

The IMP proved to be a valuable tool in the planning and development of the Budd app. This study provides researchers and practitioners with valuable information that may help them to set up their own health interventions.

**International Registered Report Identifier (IRRID):**

RR1-10.2196/39678

## Introduction

### Background

Chemsex is defined as the intentional use of drugs before or during sex among men who have sex with men (MSM) to extend and intensify sexual sessions [1]. Chemsex could include a variety of drugs [1-3]. The use of ecstasy, cocaine, crystal methamphetamine, new psychoactive substances, γ-hydroxybutyrate or γ-butyrolactone, speed, and ketamine is mainly described in a chemsex context in Belgium and the Netherlands [4-6]. Chemsex sessions can last several days, usually involve multiple sexual partners, and are primarily organized at people’s (private) homes [2]. A systematic review reports that the prevalence of chemsex varies from 3% to 29%, depending on the definition and setting [7]. Throughout this paper, in an effort to improve readability, we will use the term *chemsex participants* to refer to MSM who engage in chemsex.

Over the past decade, this combination of sexual behavior and the use of illicit drugs has been the subject of increasing research [8,9]. Although chemsex is associated with a range of sexually and psychologically beneficial outcomes such as increased sexual confidence, a greater desire for sex, increased sexual pleasure, and stronger feelings of intimacy and closeness [10,11], there is also extensive research that links chemsex with a wide range of high-risk behaviors and consequent physical, psychological, and social health harms [12,13]. These harms, which are comprehensively described further in this paper, include increased risk of HIV and sexually transmitted infection (STI) transmission [14,15]; anxiety and depression [16,17]; poor performance at work [2,18]; drug dependence; and drug overdose [19,20], with reports of unconsciousness and death [21,22].

These negative impacts pose a public health challenge for health professionals [9,23]. It is important to understand how interventions can be used effectively to reduce the potential impact of high-risk behavior in this key population. Of note, intervention programs that deal specifically with chemsex are scarce [21,24]. MSM who engage in chemsex and who look for care and support often do not access the specialized help they need [6,25]. Traditional drug counseling services usually have insufficient knowledge of sexual health problems, whereas sexual health clinics often lack expertise on substance use [26]. In addition, chemsex participants seem to seek knowledge about harm reduction practices mainly on the web or within the community as opposed to consulting health care providers [27]. Easily accessible evidence-based interventions should therefore be set up alongside traditional counseling services to promote harm reduction.

### Goal of the Project

On the basis of these findings, we launched the *Chemified project* in September 2019. This project was initiated to address the lack of evidence-based support tools and the current information and care needs of chemsex participants [6]. The ultimate goal of the project was to develop a mobile health (mHealth) intervention (named *Budd*) to support and inform chemsex participants, reduce the negative impacts associated with chemsex, and encourage more reasoned participation. The rationale for choosing an mHealth intervention is described in more detail in a dedicated article [28]. In short, on the one hand, mobile apps are considered to be facilitators of chemsex, thereby contributing to the health problem [28]; for example, geospatial dating apps are used to search for sexual partners, keep each other informed about chemsex sessions, discuss drug- and sex-related preferences, and exchange information regarding the availability or possibility of purchasing drugs [2,29-31]. On the other hand, mobile apps can be part of the solution. People who participate in chemsex already make frequent use of web-based resources; therefore, they are already familiar with how they work [32]. In addition, for many people, a smartphone is a communication tool that is permanently switched on, which makes it possible to provide support and care when and where they are needed, including during chemsex sessions.

## Methods

### Overview

This paper describes the development of the Budd app using the intervention mapping protocol (IMP) [33]. The IMP is a systematic approach that aims to support the development of theory- and evidence-based health promotion interventions [33]. The method increases both effectiveness and efficiency through an iterative process of evidence review, application of theory-based strategies, and consultation with stakeholders [33].

The IMP has already been successfully applied to develop a range of eHealth programs for several health behaviors such as a healthy diet [34,35], physical activity [36], alcohol consumption [37], sexual health [38,39], mental health promotion [40,41], and cyberbullying [42].

**Figure 1:**
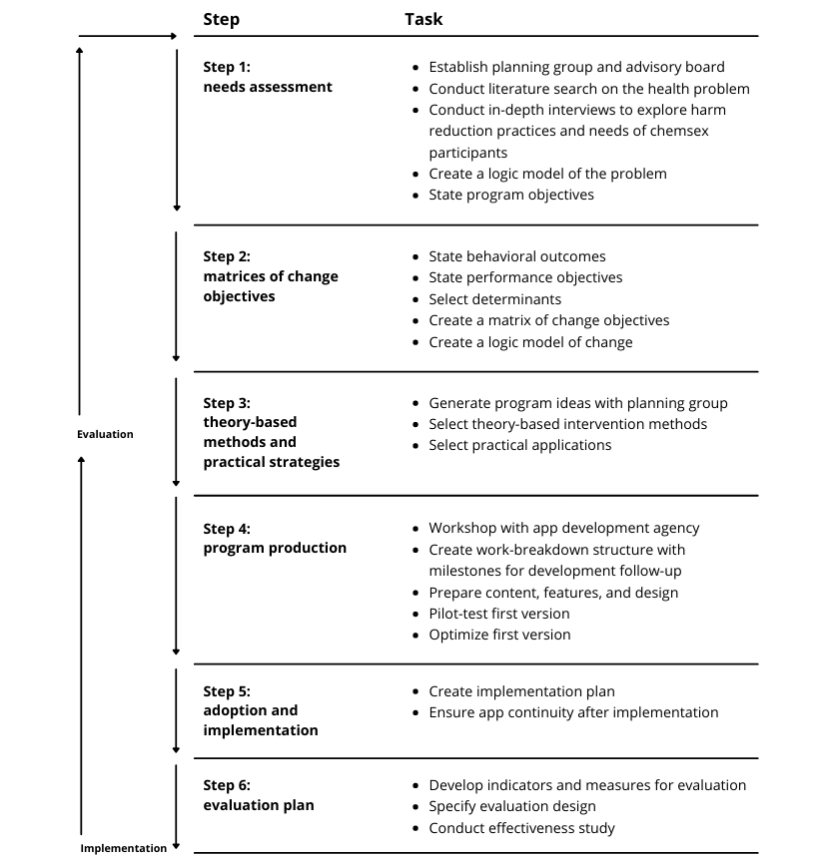
Intervention mapping steps applied to the Chemified project.

The IMP consists of 6 steps to be taken to develop, implement, and evaluate health promotion interventions [33,43]. In each step, the program designer applies findings from evidence, theory, and their own research. When the tasks in 1 of the 6 steps are completed, it serves as a starting point for the next step [33]. This paper focuses on the first 4 steps of the IMP. A separate article will be dedicated to the effectiveness study and implementation of the intervention. The application of each of the first 4 steps (Figure 1) is outlined and described in the following sections.

### Step 1: Needs Assessment—Logic Model of the Problem

#### Task 1: Establish Planning Group and Advisory Board

A planning group and an external advisory board were established at the start of the project in September 2019. The planning group was a collaboration between the Institute of Tropical Medicine (ITM) and the University of Antwerp and consisted of 5 people: a mental health scientist and sexologist with expertise in supporting chemsex participants (ITM), head of the HIV and STI outpatient department of ITM, a professor of *Health Communication* and *Media Uses & Effects* (University of Antwerp), a professor of *Strategic Communication and Persuasive Technology* (University of Antwerp), and a junior researcher with a background in sociology and experience in the development of mHealth apps (ITM).

The project benefited from the different partners’ combined expertise in health communication and technologies, persuasive communication, and technologies with regard to chemsex participants. The first author (CH) scheduled individual or planning group meetings with group members on a weekly basis to discuss the progress of the project and obtain advice. These meetings took place regularly to ensure continual evaluation and timely adjustment.

A group of key stakeholders served as an external advisory board. This group consisted of professionals from the Flemish center of expertise on alcohol, illegal drugs, and psychoactive medication and the Flemish expertise center for sexual health (Sensoa).

Potential app users were engaged in all phases of intervention planning. The reason for involving them at each decision point was to increase the probability of designing an intervention that is more likely to be effective and accepted because their involvement ensures that the intervention best meets the needs of the target group and the context in which it will be used [43].

#### Task 2: Conduct a Needs Assessment to Create a Logic Model of the Problem

##### Overview

A needs assessment was performed between September 2, 2019, and March 31, 2020. First, existing literature was consulted to analyze the health problem of *participating in chemsex* to get a clear picture of the associated health risks and risk behaviors. Second, the possible determinants of each of the risk behaviors were identified through in-depth interviews and behavior change theories (BCTs). Our intervention did not aim to include determinants on the environmental level (eg, health care providers and peers), which is why only determinants on the individual level were identified.

##### Literature Search on the Health Problem and Risk Behavior

The literature search involved articles retrievable via the PubMed, ScienceDirect, and Google Scholar databases. The following search terms were used: “chemsex,” “drugs,” “risks,” “health,” “behaviour,” “motivation,” “MSM,” “sexualized drug use,” and “men who have sex with men,” as well as combinations of these terms. Relevant papers were selected and reviewed. In addition, the reference lists of the selected articles were reviewed manually to identify other relevant articles.

##### Analysis of Behavioral Determinants: Personal Factors

Intervention mapping uses a multitheory approach because different theories may focus on specific aspects of behavior or behavior change [43,44]. The BCTs we selected to guide the selection of determinants for our intervention were the social cognitive theory (SCT) [45,46], the theory of planned behavior (TPB) [47,48], and the precaution adoption process model [49]. The rationale for choosing these BCTs is based on existing literature and described in the *Results* section.

After analysis of the problem, data were collected through 20 semistructured in-depth interviews with Flemish chemsex participants [6]. The aim of these interviews was to gain more insight into the specific Flemish chemsex context and also complement the results of the analysis of the problem by exploring the determinants of chemsex behavior and the needs of the key population. The interview guide explored current risk reduction practices (before, during, and after a chemsex session) and the needs of chemsex participants. Specific methods, participant characteristics, and detailed results have been published in a dedicated article [6]. The results of the interviews enabled us to assign relevance to the health risks of MSM engaging in chemsex and the underlying determinants. The results also helped us to formulate achievable performance objectives (step 2 of the IMP) based on the preventive measures MSM take when participating in chemsex.

#### Task 3: State Program Objectives

The information gathered from this first step results in a logic model of the problem [33]. On the basis of this model, the planning group selected the program objectives.

### Step 2: Matrices of Change Objectives—Logic Model of Change

The tasks in step 2 (Figure 1) were completed through consulting existing literature, expert opinion of the planning group, results of the in-depth interviews, and BCTs. First, behavioral outcomes were described. These were derived from the logic model of the problem to achieve the program objectives identified in the previous step. Second, these behavioral goals were further operationalized into performance objectives, which are specific goals that define who and what will change as a result of the intervention [33]. Third, factors that influence behavior, referred to as determinants in intervention mapping terminology, were identified for each performance objective [33]. Finally, the behavioral objectives, associated performance objectives, and determinants were formulated and presented in a matrix. A logic model of change was created to summarize these outcomes.

### Step 3: Theory-Based Methods and Practical Strategies

In step 3, theoretical methods were selected that have an impact on each behavioral determinant [33]. These behavior change methods (BCMs) are conceptualized as a process of change derived from theory [43]. Next, the BCMs were turned into practical applications (PAs). An application is the specific strategy in which the methodology is expressed in the intervention [50].

To successfully carry out this step, a list was created of all BCMs that were predicted to achieve the change objectives. We used BCTs and results from empirical research to establish this list. After a detailed discussion with the planning group, we reduced the long list of potential BCMs into a short list, and these BCMs formed the building blocks of the first version of the Budd app.

At this stage of the IMP, one often goes back and forth between steps 3 and 4 [33]; for example, we adjusted the choice of certain BCMs and PAs after carrying out a pilot study of the first version of the Budd app (carried out in step 4; refer to the *Task 2: Carry Out Pilot Study* section).

### Step 4: Program Production

In step 4, the selected PAs were organized and built into a coherent intervention [43].

#### Task 1: Define and Develop Structure, Components, and Content

A workshop with the planning group and the app development agency marked the start of the actual development process. Three goals guided this workshop: introduce the app developers to the project and the potential app users, set priorities, and draw up an app development plan. The entire development process was divided into 5 milestones (Multimedia Appendix 1). Each milestone was assigned a time frame that had to be approved by the planning group and adjusted by the app development agency as necessary. The first version of the Budd app was developed in December 2020.

#### Task 2: Carry Out Pilot Study

##### Study Design and Respondent Recruitment

To evaluate the first version of the Budd app, we performed a mixed methods usability evaluation. Think-aloud testing was followed by administering a usability questionnaire and completed by conducting semistructured interviews after an app testing period of 2 weeks. Our objectives were to (1) identify design, usability, and functionality issues; (2) assess acceptability; and (3) assess satisfaction with the intervention components.

We collected feedback from 8 chemsex participants, of whom 4 (50%) were recruited from the respondents who participated in the in-depth interviews (step 1) and ticked the box on the informed consent form indicating that, at a later stage, they would like to provide feedback on the developed intervention. The remaining (4/8, 50%) respondents were recruited through pre-exposure prophylaxis (PrEP) or HIV and STI consultation at ITM.

The eligibility criteria included being aged at least 18 years; self-identifying as a male member of the lesbian, gay, bisexual, transgender, queer, and similar minority community; being able to understand, and express oneself in, Dutch or English; having intentionally used drugs to have sex within the past 12 months; and owning a smartphone.

##### Procedures

The study was conducted at ITM by CH between February 1, 2021, and April 30, 2021. At the start of the study, the procedure was explained, and the participants signed the informed consent form. The researcher helped each participant install the app on their smartphone. First, participants completed a preset list of different tasks of varying levels of complexity that cover the full range of functionalities of the app (Multimedia Appendix 2). The researcher was not allowed to help participants complete the tasks. To evaluate usability, the think-aloud method was applied to understand respondents’ thoughts as they occurred and attempted to cope with the issues they encountered [51]. After performing all of the tasks, the study participants filled out the 10-item system usability scale (SUS) questionnaire [52]. The SUS is a widely used validated method for assessing the usability of a system [53]. Next, the study participants tested the Budd app in their own environment for 2 weeks. After this test period, a follow-up interview took place at ITM to assess the (1) design, (2) usability, (3) satisfaction with the included intervention components, and (4) acceptability. The interview guide can be found in Multimedia Appendix 3.

##### Data Analysis

A pragmatic analysis approach was used to analyze the usability testing and follow-up interviews [54]. In both exercises, the researcher took extensive notes [55,56]. This method was chosen to save time and resources in data processing and subsequent analysis [56]. During the usability test, the researcher collected data by observing the study participants carrying out the tasks while also writing down the thought processes and feedback they shared. During the usability test as well as the in-depth interview 2 weeks later, notes were categorized in a template with subject headings created beforehand [57]. For the usability test, the notes were structured into the categories *usability issues* and *ideas for improvement*. The usability issues were further specified for each intervention component. For the follow-up interview, the researcher registered the main outcomes for each of the 4 topics by writing a summary for each topic discussed. Notes from both studies were analyzed by conducting a deductive thematic analysis [58].

After completing the tasks, all participants completed the SUS questionnaire. The scale consists of 10 questions measured using a 5-point Likert scale (ranging from strongly disagree to strongly agree) and is based on 3 usability criteria: effectiveness, efficiency, and user satisfaction [59]. The final score from the SUS can range from 0 to 100 points. A score of >68 points is above average and indicates adequate usability. In this study, the individual SUS scores of the participants were summed and then divided by the number of participants to obtain a mean usability SUS score.

#### Task 3: Optimizing the Intervention

The pilot study allowed for the collection of valuable feedback, and these results were then translated into concise modifications and additions to the first version. The second version of the Budd app was finalized in November 2021.

### Ethics Approval

Appropriate ethics approval was granted by the ITM institutional review board on December 6, 2019, for the in-depth interviews (1344/19) and on December 15, 2020, for the pilot study (1442/20). Informed consent was obtained from all participants.

## Results

### Step 1: Needs Assessment—Logic Model of the Problem

#### Task 1: Establish Planning Group and Advisory Board

A planning group with research partners, an advisory board with stakeholders, and a group of potential users have been involved and consulted from the start of the project.

#### Task 2: Conduct a Needs Assessment to Create a Logic Model of the Problem

##### Literature Search on the Health Problem and Risk Behavior

We found that *participating in chemsex* is associated with a wide range of health risks and related risk behaviors. Although the majority of chemsex participants do not identify with problematic use and experience fewer minor consequences [10,11], there is a group of chemsex participants who do experience harms resulting from their participation. The health problems associated with chemsex can largely be divided into 4 categories. Each category is explained in more detail in the following sections.

The first cluster of chemsex health problems cited in the literature involves drug-related physical harms. An acute risk, frequently reported, is drug overdose [8,60]. The degree of overdose and its effects can range from aggression, heart palpitations, breathing difficulties, panic attacks, delusions, hallucinations, dehydration, overheating, memory loss, and unintentional injury to loss of consciousness and even death [8,60]. Depending on the type of drug, there are also many potential long-term risks, including substance dependency, alterations of cognitive functions, heart disease, psychosis, high blood pressure, movement disorders, and weight loss [2,61].

The risk behaviors contributing to these health problems are a variety of high-risk drug use practices. Polydrug use is the norm for many MSM engaging in chemsex, although percentages vary between 7% and 78.3%, depending on the study locations and definition used (starting from 2 or 3 substances) [2,13,62-64]. Other important risk behaviors include the use of crystal methamphetamine [29,65] and new psychoactive substances [66], injecting drug use during sex (known as “slamming”) [18,29,67,68], sharing user equipment (snorting devices and injecting equipment) [18], using high doses [2], extensively redosing [2,69], and going multiple days without sleep [67].

The second cluster of reported chemsex health problems is related to sexual health. Taking drugs before or during sex helps MSM to experience sex without inhibitions [70,71]. This ensures that people are prepared to participate in a wide range of sexual acts, which sometimes go beyond their limits [2]. Chemsex has been associated with high-risk sexual behaviors, including group sex; condomless anal intercourse; fisting; bondage, discipline, sadism, and masochism; having a high number of sexual partners; long sexual sessions that can lead to rectal and penile trauma; and transactional sex [2,7,18,26,63,66,72,73].

MSM who engage in chemsex are more likely to contract HIV, hepatitis C, and other STIs than those who do not engage in chemsex [18,62,74]. Taking drugs can also lead to sleep problems and memory impairment, which, when combined with prolonged sex sessions, can cause people to forget about taking antiretrovirals for HIV management or PrEP [75]. Certain drugs have also been shown to interact with antiretroviral drugs, which is reflected in a negative impact on clinical HIV outcomes [75].

Chemsex participants may also experience difficulty in having sex without drugs because the drugs used hugely increase sexual drive and desire [2]. In this context, being unsatisfied with one’s sex life is associated with participating in chemsex [60]. The strong feelings of intimacy and connection experienced during a chemsex session disappear after the drugs wear off, contributing to the level of dependency in which a large proportion of chemsex participants are in search for connection [76]. Some chemsex participants also report being the victim of nonconsensual sex while under the influence of drugs [2,60]. A recent study among MSM in Amsterdam showed that 41.4% of the participants who engage in chemsex reported passing out and not remembering what happened while under the influence of drugs [77].

The third cluster of chemsex health problems being increasingly reported in the literature involves the adverse impact of chemsex on mental health [17,78,79], although research on the topic remains limited. A recent systematic review found a positive association between chemsex and depressive symptoms, anxiety, and dependence on drugs or other substances [80]. The practice of slamming, especially, is associated with a greater number of mental health symptoms [29,66].

The acute mental health risks of participating in a chemsex party are caused by the substances used. Depending on which drugs are used, risks can include agitation, confusion, feelings of anxiety, aggression, restlessness, and irritability [2]. With crystal methamphetamine specifically, there is a risk of extreme paranoia and panic attacks [29,70]. People often experience poor concentration, difficulty with information processing, and feelings of anxiety and irritation in the days after a chemsex party (*comedown*) [2,6]. These feelings are a result of using stimulant drugs (crystal methamphetamine, ecstasy, and mephedrone) [81]. In the long term, drug use can also lead to anxiety disorders, depression, psychosis, memory problems, and personality changes [2,81].

Anecdotal evidence at our clinic at ITM further indicates an impact on other areas of chemsex participants’ lives. Some state that they struggle with relational, financial, and work-related factors as a result of their participation in chemsex. There is limited evidence fully identifying the social impact that chemsex behaviors have on chemsex participants’ lives. In a study among chemsex participants in Dublin, 25% of the participants reported that chemsex had a negative impact on their lives [15]. The impact on the work situation seems to be most pronounced during the *comedown* period because people experience difficulties in working efficiently, concentrating, and feeling motivated. As a result, chemsex participants are more likely to perform poorly at work or report sick on the days after a chemsex party [2,18]. Chemsex participants who use methamphetamine report that the use of the drug reduces their ability to fulfill daily tasks [82].

##### Analysis of Behavioral Determinants: Personal Factors

The determinants of chemsex participation were identified and categorized on the basis of a literature review guided by the selected BCTs and in-depth interviews.

Awareness is defined as the degree of knowledge and understanding of one’s own unhealthy behavior [49]. The findings from the interviews indicate that the effects of drugs can greatly reduce the awareness of one’s own risk behavior during a chemsex session [6]. Respondents report losing themselves completely in the intense feelings of sexual arousal and disinhibition [6]. Only when a person is also aware that they are engaging in risk behavior (eg, know how much time they leave between 2 dosages and know their own [sexual] boundaries) can they consider changing this behavior. Awareness of one’s own risk behavior is seen as an essential first step in the process of behavior change, according to the precaution adoption process model [49]. In an attempt to increase awareness, we included this essential determinant in the development of our intervention.

The transfer of knowledge is often a key element in traditional health education [50]. Information about health behavior and the health risks of certain behaviors is necessary to enable behavior change. The in-depth interviews strongly emphasized this need for reliable information [6]. Particular attention needs to be paid to information about chemsex drugs, the effect of combined chemsex drugs, how best to act in the event of an emergency, and an overview of existing (drug and sexual) health care services. Respondents indicated that a lack of information often hindered them in adopting risk reduction practices [6]. This need is also reflected in studies conducted in other countries, such as a UK study that showed a need for reliable and nonjudgmental information about safe drug practices [8] and the European Men-Who-Have-Sex-With-Men Internet Survey 2017, which found that respondents scored low on HIV and STI transmission knowledge, postexposure prophylaxis knowledge, PrEP knowledge, HIV test and treatment knowledge, and hepatitis A and B test knowledge [83]. Knowledge is thus a presumably important determinant in practicing safer chemsex.

According to the SCT, human behavior is, to a large extent, determined by the expectations one has of a behavior [46]. One of these expectations is the person’s belief about their ability to successfully influence their environment [46]. This concept of *self-efficacy* is a central concept in the SCT, the importance of which is widely recognized. In the context of our intervention, the SCT is cited in several studies as a suitable model for behavior change among individuals who take drugs. Numerous studies have shown that self-efficacy plays an important role in abstinence, substance relapse, and adopting harm reduction measures [84,85]. More specifically, a UK study shows that engaging in chemsex is associated with lower sexual self-efficacy [86]. A study among MSM who had tested positive for HIV and who were using methamphetamine shows that being convinced to be able to say *no* to drugs (*drug assertiveness skills*) was associated with reduced frequency and amount of drug use as well as less sexual sensation seeking and unprotected sex [87]. Higher self-efficacy also has a direct positive effect on HIV treatment adherence [88,89] and PrEP adherence among MSM in the United States [90].

The TPB states that the best way to predict behavior is to ask people whether they intend to exhibit that behavior: the behavioral intention [47,48]. According to Fishbein and Ajzen [47], this behavioral intention is determined by 3 factors: the person’s own views (attitude), the views of others (subjective norm), and the estimation of the person’s own possibilities of carrying out the behavior (perceived behavioral control, based on the concept of *self-efficacy* mentioned previously).

The TPB is one of the most widely applied behavior change models and has been shown to be effective in explaining and predicting a wide range of health behaviors [91]. Several studies report that the TPB is an appropriate model for explaining, understanding, and predicting a variety of chemsex-related health behaviors, some of which we identified in step 1 of the IMP. These include safer sex behaviors [92], condom use [93,94], therapy adherence [95], intention to enter drug and alcohol treatment [96], and the use of safe injecting procedures [97]. Furthermore, a meta-analysis supported the use of the TPB as a valuable framework for designing interventions to reduce heterosexual risk behavior [98]. Therefore, we considered the TPB suitable to guide us in the development of our intervention.

#### Task 3: State Program Objectives

**Figure 2:**
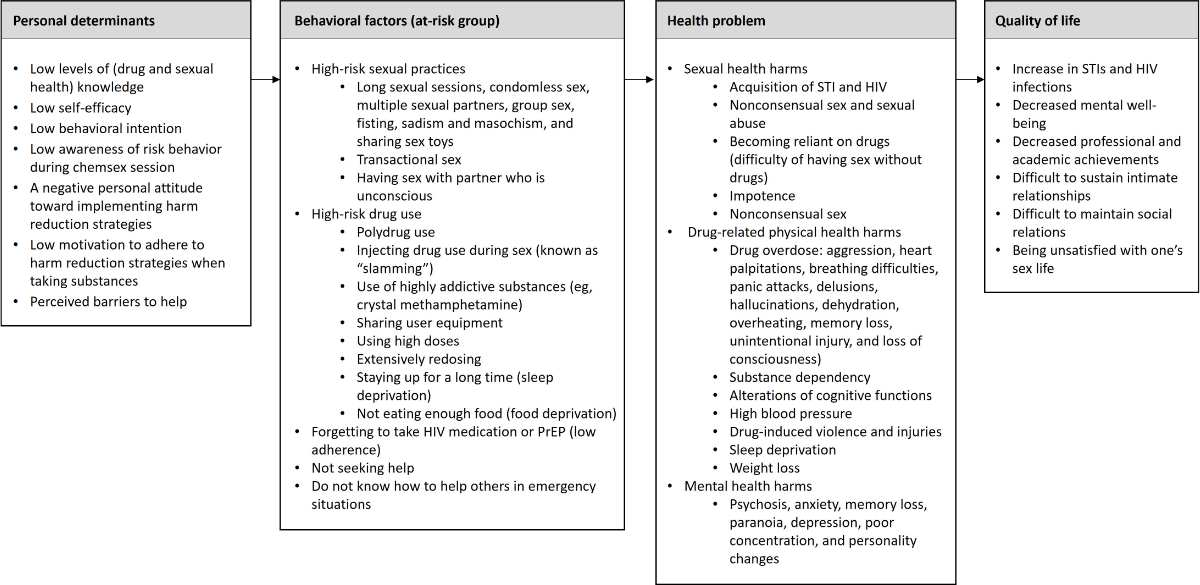
Logic model of the problem. PrEP: pre-exposure prophylaxis; STI: sexually transmitted infection.

We created a logic model of the problem based on the information gathered in this step (Figure 2).

The results of the interviews helped to assign relevance to the health harms and risk behaviors. On the basis of this information, the planning group selected five program objectives: (1) increase safer drug use, (2) improve planning and monitoring of participation in chemsex sessions, (3) facilitate access to health care and support, (4) increase therapy compliance (HIV medication and PrEP), and (5) enhance assistance of other participants during a chemsex session.

### Step 2: Matrices of Change Objectives—Logic Model of Change

**Figure 3:**
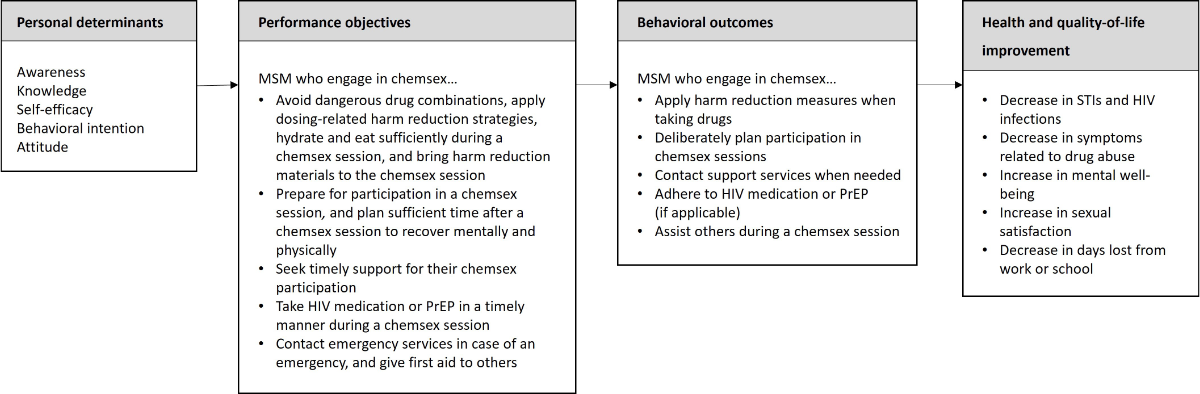
Logic model of change. MSM: men who have sex with men; PrEP: pre-exposure prophylaxis.

On the basis of the program objectives, we formulated 5 behavioral outcomes for the intervention (Figure 3). For all 5 subbehaviors, separate matrices of change were created (Multimedia Appendix 4).

### Step 3: Theory-Based Methods and Practical Strategies

**Figure 4:**
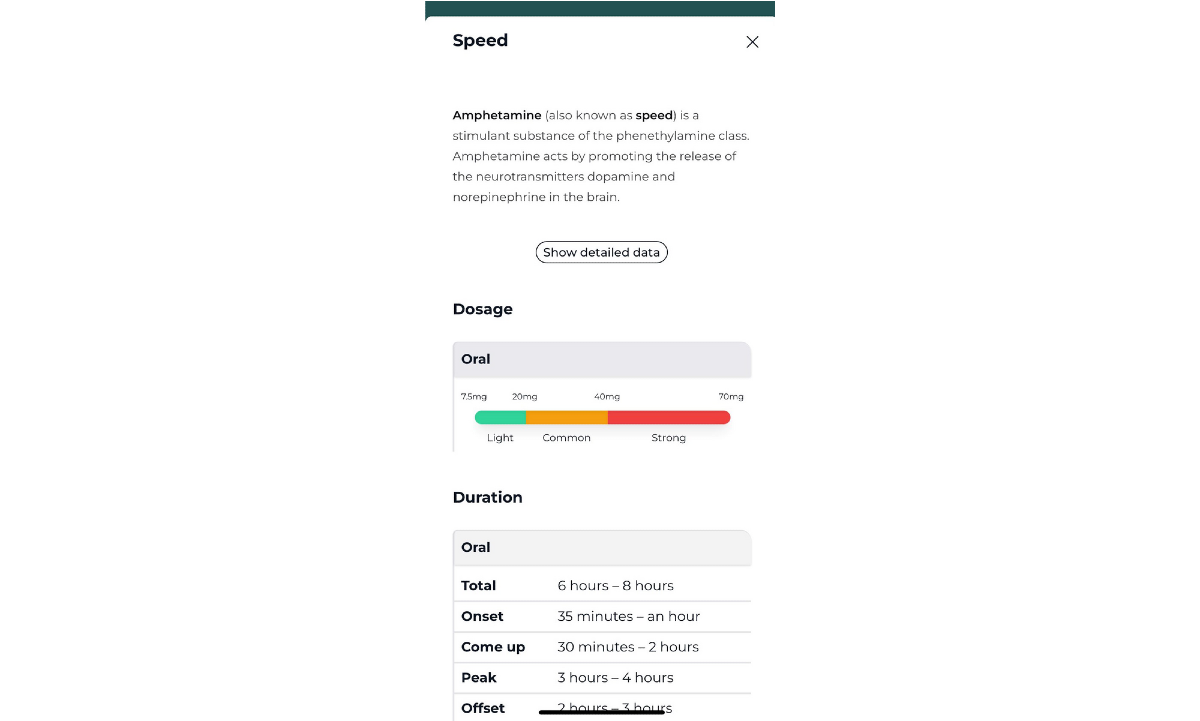
Practical application 1: drug information.

**Figure 5:**
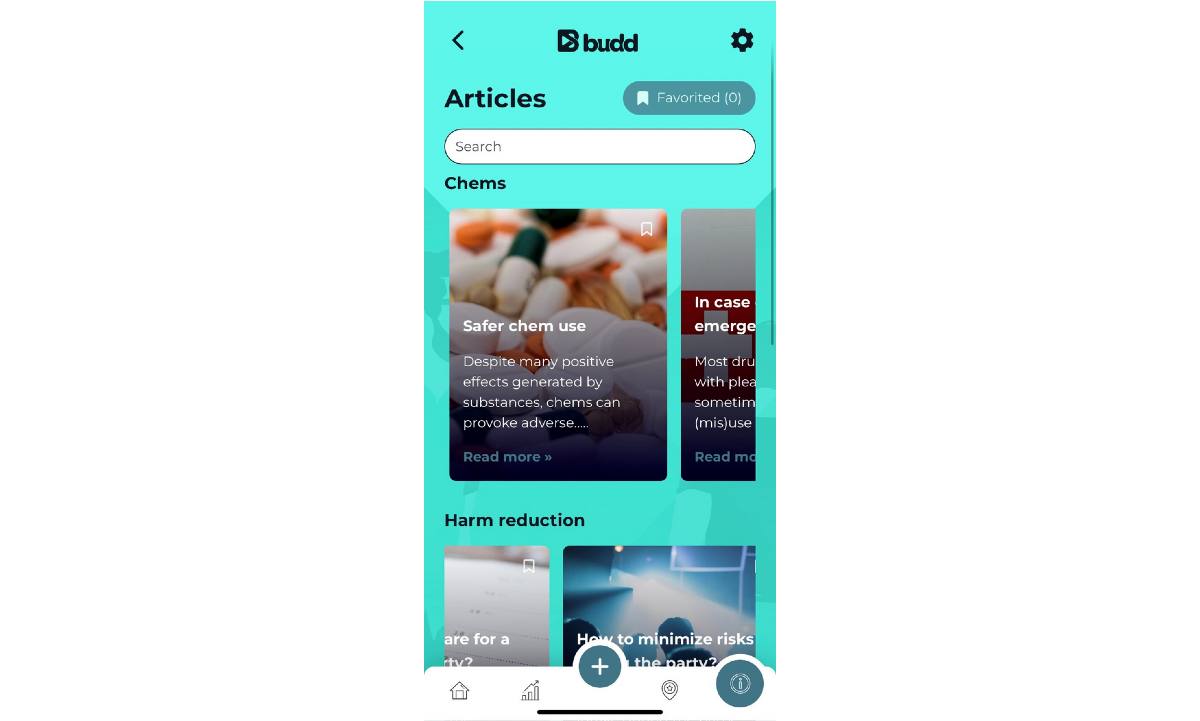
Practical application 3: articles about chemsex-related topics.

**Figure 6:**
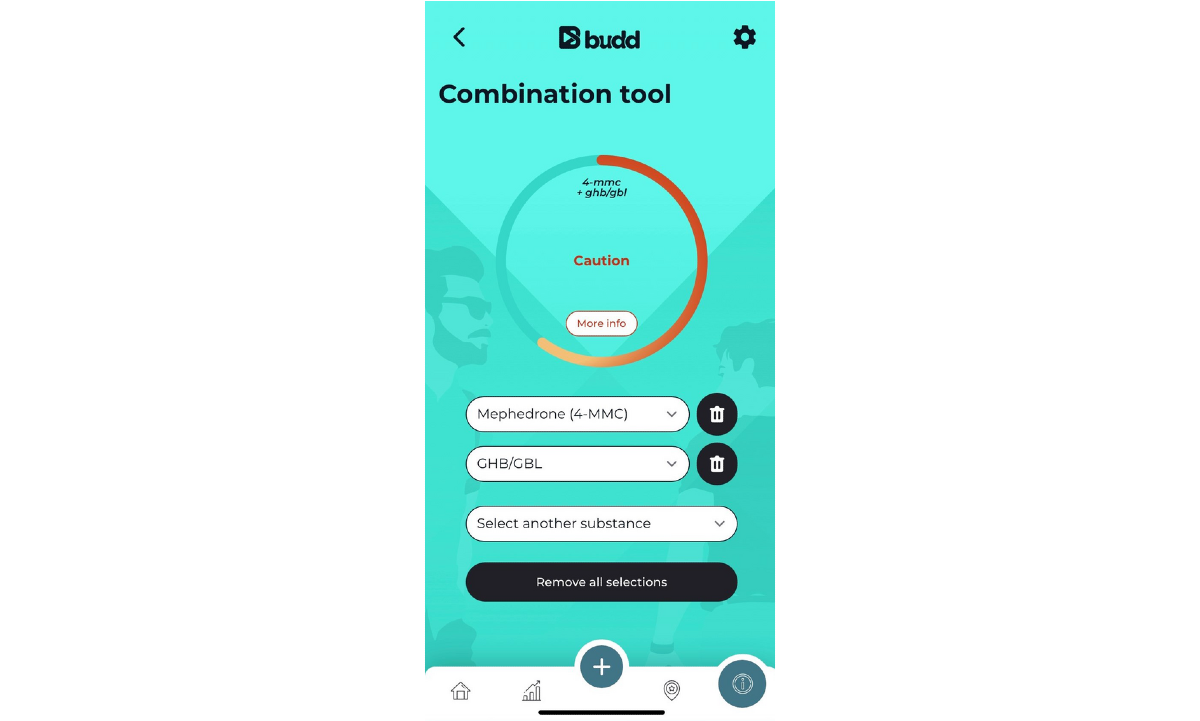
Practical application 2: drug combination tool.

Multimedia Appendix 5 lists the BCMs and PAs used in the Budd app; for example, “providing information” is used as the main BCM to increase the user’s knowledge, attitudes, and behavioral intentions [33]. A relevant theory for knowledge transfer is the elaboration likelihood model [99]. This theory states that people are more likely to think about a particular information message than others depending on the situation or context. According to this model, changes resulting from central information processing are more stable, more persistent, and more predictive than those resulting from peripheral processing. People often do not process information centrally because they do not have time, they are not motivated, the information is too difficult to understand, and so on. With the Budd app, we try to promote this central processing of information. Established methods to stimulate this are active learning, participation, and personal relevance, often in combination [99]. To ensure the relevance of the included information and to match the users’ beliefs as much as possible, we created the initial version of the app based on the results of the interviews and made final adjustments based on the results of the pilot study. The Budd app contains 7 PAs based on the BCM of “providing information.” The user receives both general information (eg, PA1: drug information about commonly used chemsex drugs [Figure 4], PA3: articles about chemsex-related topics such as “Safer chem use,” “STIs and chemsex,” and “Chems and consent” [Figure 5], PA4: an overview of health care and support in Flanders (the Flemish region of Belgium), and PA5: emergency information) and more tailored information (eg, PA2: assessing the interaction of drugs the user takes during a chemsex session using the drug combination tool [Figure 6] and PA6: testimonials from other MSM who participate in chemsex). A knowledge quiz (PA7) with feedback per completed question was also integrated into the app to promote central information processing.

**Figure 7:**
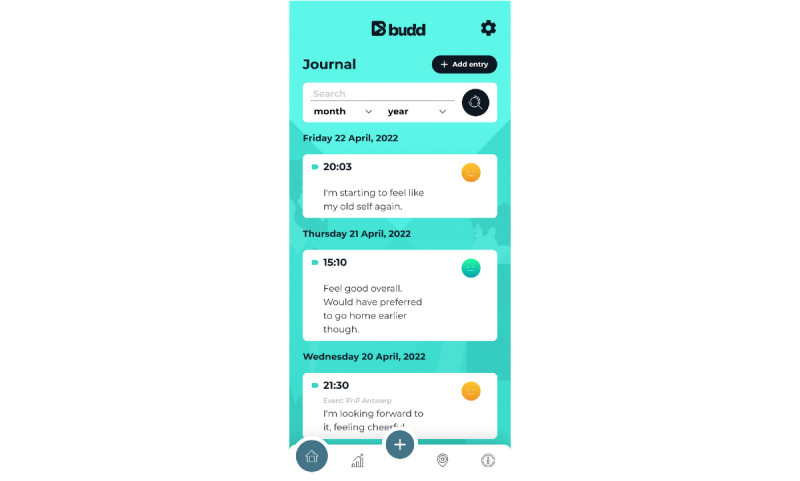
Practical application 10: journal.

**Figure 8:**
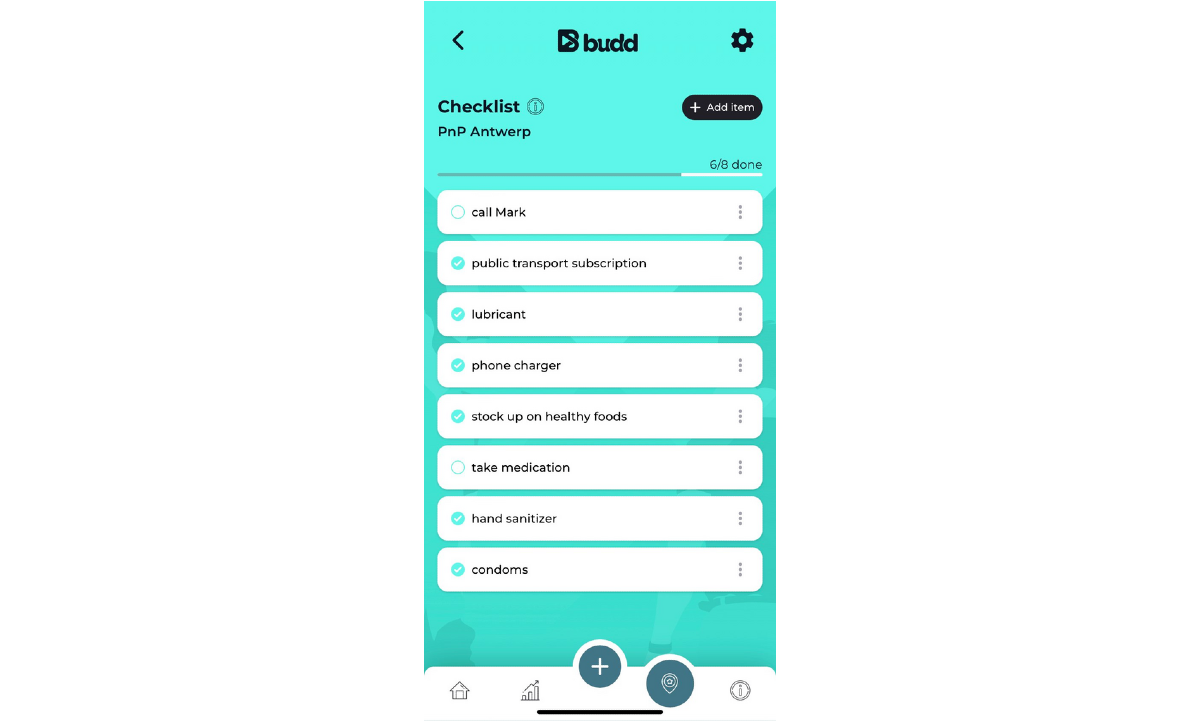
Practical application 11: personal checklist.

**Figure 9:**
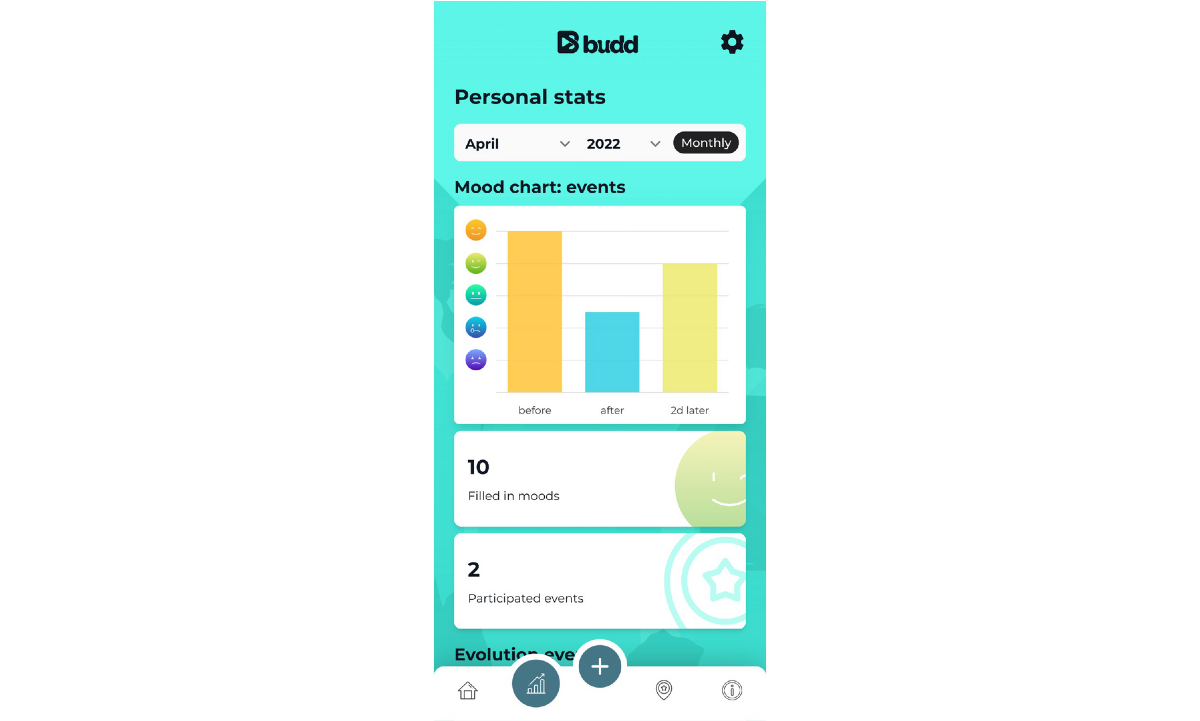
Practical application 13: personal statistics page.

Another example is the BCM “self-monitoring of behavior” to increase impact on the determinant *awareness*. This method is widely used in health interventions and has been found to be effective for a variety of health behaviors [100-102]. Self-monitoring encourages users to record their behaviors and gain insight into their chemsex participation. We have translated this method into the following 6 PAs:

- PA8: a mood survey related to participation in chemsex sessions (at check-in, check-out, and 2 days later)
- PA9: a notebook with time stamps to allow the user to monitor drug intake (especially useful for monitoring gamma-hydroxybutyrate and gamma-butyrolactone intake) during the chemsex session
- PA10: a journal where reported data are centralized (Figure 7) and which can also be used to make daily entries
- PA11: a personal checklist related to each planned chemsex session where the user can list to-do items or materials to bring with them (Figure 8)
- PA12: reflection on answers to the questions from the preparation tool (2 days after the chemsex session)
- PA13: a *personal statistics* page that contains a visual representation of mood, number of chemsex sessions participated in, evolution of chemsex sessions per month, and number of hours spent on average at a chemsex session (Figure 9)

### Step 4: Program Production

#### Task 1: Define and Develop Structure, Components, and Content

**Figure 10:**
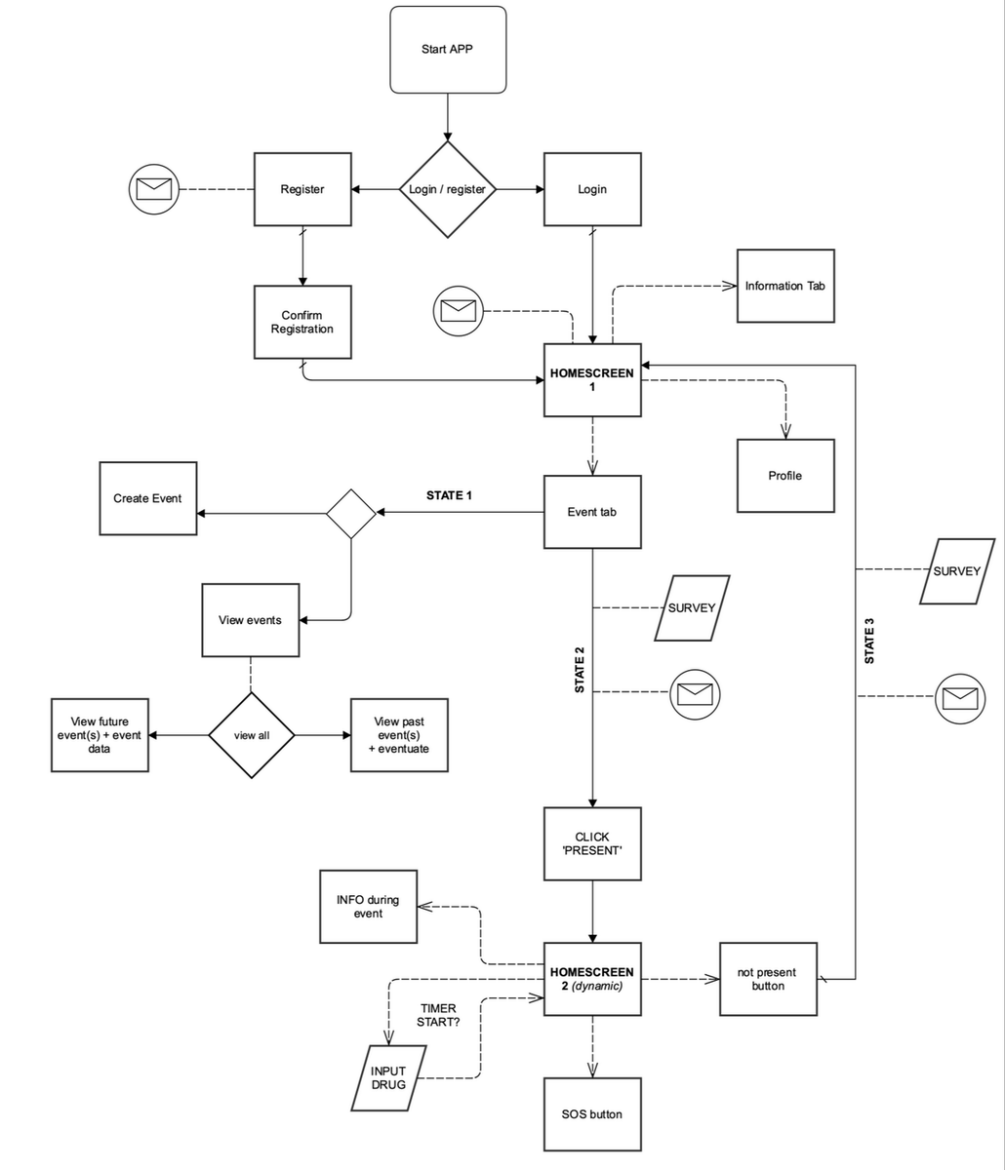
First user flow.

During a workshop with the planning group and app developers, we performed the Must Have, Should Have, Could Have, Won’t Have This Time exercise [103] to determine app components and set priorities. After the exercise, all possible features were classified into one of the following four categories: (1) must haves (required; otherwise, there is no workable product), (2) should haves (these requirements are very desirable, but without them the product is still usable), (3) could haves (options that will only be included if there is enough time and budget), and (4) won’t haves (will not be discussed in this phase but may be discussed in the future). On the basis of this exercise, the app developers created the first version of the user flow (Figure 10). The workshop concluded with a brainstorm session about possible app names and designs.

#### Task 2: Carry Out Pilot Study

This section discusses the results of the pilot study on the first version of the Budd app.

##### Executing a Set of Tasks

The participants performed various tasks within the Budd app, providing us with a rich data set containing their feedback. The usability issues and ideas for improvement mentioned by the respondents were classified according to their intervention components. All results can be found in Multimedia Appendix 6.

##### SUS Score

The average usability score was 76.88, which indicated the need to make only minor usability improvements to the first version.

##### Follow-up Interview

Respondents used the app for different purposes; for example, some (4/8, 50%) of them found the informative articles especially useful because they contained practical, to-the-point, and clearly defined information. Of the 8 respondents, 2 (25%) found the process of planning chemsex sessions helpful because it increased awareness of participation before attending a session, whereas 2 (25%) found this component to be less meaningful because they usually do not know in advance when they will participate in a chemsex session as these sessions happen unexpectedly more often than not or at the last minute.

Respondents also provided feedback on potential improvement of the app components. Some (5/8, 63%) of them mentioned that basic information on different substances was lacking, whereas others (2/8, 25%) stated that information on specific new psychoactive substances and the risks of slamming was missing. Respondents also stated that the section on drug combinations could be worded more in layman’s terms. When checked in on the app at a chemsex session, respondents found it inconvenient that they could not easily return to the rest of the app because they had to check out first.

Finally, respondents also shared ideas for adding intervention components. These focused mainly on expanding the substance-related–information section, giving a voice to other chemsex participants, adding ways to keep track of thoughts, and increasing awareness of time during a chemsex session.

The main usability issues had already been addressed in the usability test (“Performing tasks”). The answers to the questions about usability were therefore rather limited. The 2-week test period did lead to some suggestions to improve usability that were not noticed during the first test. These included the following: it is inconvenient that there is no chemsex session overview, public holidays are not visually indicated in the calendar view, have the weeks start on Monday when “adding a new event,” and the red bar bearing the legend “Call emergency service” overlaps with text at the bottom of the screen.

Three factors were surveyed to assess acceptability: frequency of use, whether people expect to continue using the app (*intention to use*), and whether they would recommend the app to others. The frequency of use reflects the same finding as the preference for certain intervention components. Some (2/8, 25%) of the respondents only used the app just before and during a chemsex session, whereas 13% (1/8) used it primarily to read all the information, and 38% (3/8) used the app very regularly for the information it provides and to support their participation in chemsex. All respondents stated that they intend to continue to use the app when it is generally available. The reasons for this were varied and included the following: will use it (1) depending on frequency of participation, (2) because it has a scientific basis, (3) to monitor substance use, (4) to monitor participation in chemsex, (5) as a personal safety tool, and (6) for the educational aspect. All respondents were also willing to recommend the app to others but again for different reasons: to (1) prepare people just starting to participate in chemsex, (2) make people more conscious about their participation in chemsex, and (3) inform people about assessing the interaction of drugs using the drug combination tool. Of the 8 participants, 3 (38%) stated that they hoped that it would become a common tool in the community so that the use of the app during parties would become easier.

In general, the respondents were very positive about the design of the app. This may also be partly due to the fact that we had already carried out a small-scale design review with 5 respondents at the start of the development process. The respondents found the layout and structure to be clear. However, the respondents felt that some parts of the interface could be more consistent; for example, the placement of certain buttons and whether pop-ups should be used. The font, according to the respondents, is easy to read. The use of color was also well received, with respondents describing the colors used as neutral, calm, and generating the feeling that “we take care of you.”

#### Task 3: Optimizing the Intervention

#### Overview

After carrying out the pilot study, the results were discussed with the planning group. We prioritized the refinements and revisions of the app based on the frequency and relevance of respondent feedback and feasibility within the study period. An overview of the second scope with all changes and additions is presented in Multimedia Appendix 7.

#### The Budd App

In the following sections, we describe in brief the different components developed for the revised intervention, which went live on April 26, 2022. The Budd app consists essentially of 2 modules: an information module and individual support.

**Figure 11:**
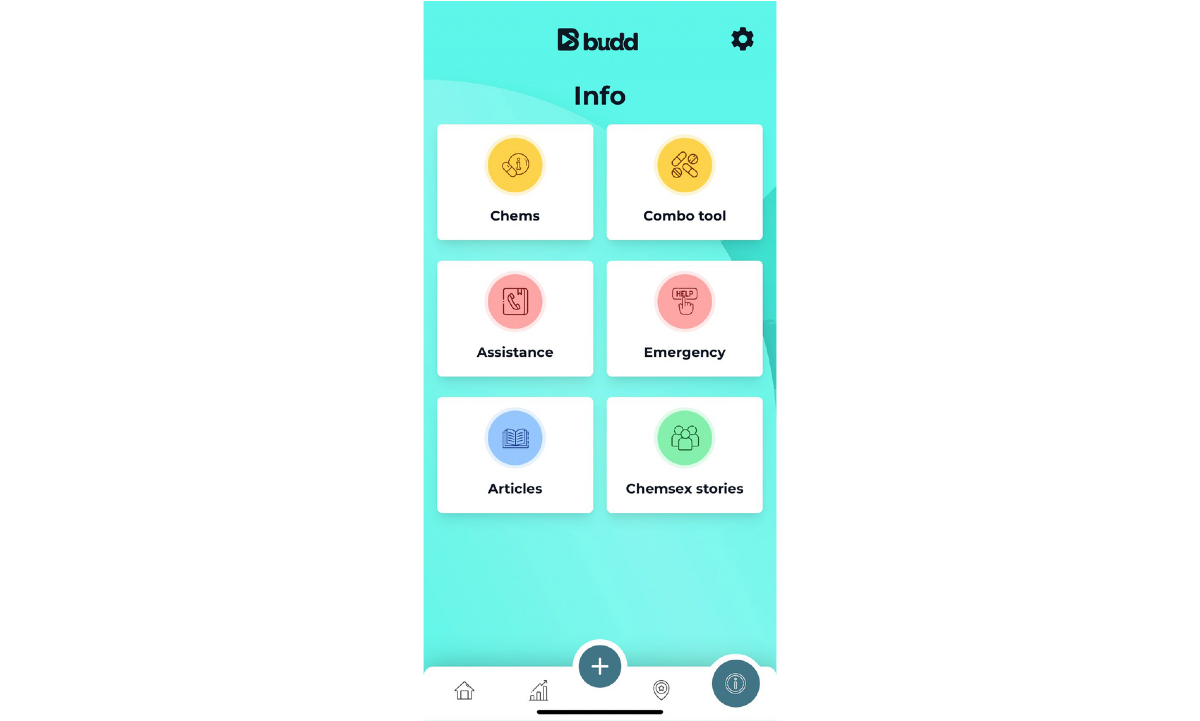
Information page.

The information module is at all times accessible to the app user. This page consists of 6 parts (Figure 11).

First, the articles cover 3 topics: chemsex drugs, harm reduction, and safer sex. Examples of articles include the following:

- “Safer chem use”
- “How to tackle the comedown”
- “TasP, PEP and PrEP: What does it mean in HIV prevention?”
- “STIs and chemsex”
- “Safe(r) slamming”

Second, the module contains “Chemsex stories,” which are testimonials consisting of other chemsex participants’ experiences. The topics include “How I handle the comedown,” “It’s not about crossing boundaries,” and “It started to affect everything in my life.”

Third, this module also contains specific information on the most commonly used drugs in the context of chemsex. For each substance, the app user will find some product information, dosage (light, medium, or strong), duration of the effects, and description of the effects.

Fourth, by using the “Combination tool,” app users can easily check the interaction among different drugs (Figure 6). The tool allows users to *combine* up to 4 different substances because we are aware that chemsex participants usually take >2 drugs during a chemsex session. At the top of this screen a circle appears whose boundary ring can fill up according to the risk and can be colored purple (low risk), light orange (caution), dark orange (unsafe), or red (dangerous). In this way, the risk can be assessed at a single glance. More detailed information on interactions among the selected substances can be found by clicking the “More info” button.

Fifth, this module contains an overview of designated local health care professionals and peer support services such as drug counseling services, sexologists, psychologists, HIV and AIDS reference centers, peer support groups, and locations where drugs can be tested to check whether they are “safe.”

Sixth and last, the information page contains an “Emergency info” button. When app users click on this button, they will find an overview of a number of emergency situations that can occur during a chemsex session and a step-by-step plan of how best to act in such situations, including overdose, heatstroke, and unconsciousness. In addition, there is a step-by-step plan, an instruction video, and an illustration of how someone can be placed in the recovery position. This page also contains a list of symptoms that indicate when a person should dial 112 (emergency services). This “Emergency info” page also contains a red button, clicking on which opens 2 buttons in the app: a (smaller) red button and a (smaller) blue one. Clicking on this smaller red button would connect the app user to 112, whereas clicking on the blue one would connect the user to his personal safety buddy. This *buddy* is someone the app user trusts and whose contact details can be added to the Budd app so that they can be easily contacted during a chemsex session.

In addition to using the Budd app to access general chemsex information, it can be used to gain a better understanding of one’s own chemsex participation and to participate in a more conscious way. To enable this, we included app components that can be of assistance to users before, during, and after participating in a chemsex session.

Via the “Events” page, app users can plan chemsex sessions in the app. The app user needs to fill in a few details such as event name, start date, end date (optional), location (optional), and notes (optional).

When a session is planned, there are 2 ways to prepare.

The preparation tool can be used to set intentions and consider certain harm reduction strategies. The tool includes an 8-item questionnaire, with questions such as “When do I intend to go home?” “How will I get home safely after the event?” “Which chems do I certainly want to avoid taking during this event?” “Have I set an alarm for my medication intake?”

Alternatively, an individualized and event-specific checklist can be completed. This checklist is a to-do list for the corresponding event. App users can add items here that they do not want to forget or certain things that they still need to organize. It is also possible to let the app suggest items such as mobile phone charger, lubricant, medication, and hand sanitizer.

When the app user has planned a chemsex session, he can check-in by moving a slider. The app now knows that the person is attending a chemsex session. If the app user unexpectedly ends up at a chemsex session, he can also click “Already at event” on the “Events” page; automatically, an event will be created. When checking in, Budd will ask the user to fill in a mood survey. This can be filled in quickly and easily by moving a bar from left to right and choosing an appropriate emoji.

Once the user has checked in at the session, the app changes to a dark mode. A stopwatch (measuring time in minutes) starts running in the menu bar to clarify that the app user is currently at a session. In this view, the checklist and the notebook can be used. The notebook sets a time stamp for each note. This allows the user to track the progress of the chemsex session and possibly monitor the time elapsed between each drug dose. The most relevant harm reduction components are shown centrally on the screen: the drug combination tool and emergency information. The other buttons of the app are still accessible in this view, but these pages are now also displayed with a dark background.

When the app user returns home, he needs to check out of the event. Budd will again ask him to fill in the mood survey. Two days after checking out (during the *comedown* period), Budd will ask this for the last time. In addition, at this time, 2 reflection questions are asked related to the preparation tool questionnaire filled in by the user before the chemsex session. By comparing the answers provided before and after the chemsex session, the app user can examine the extent to which his set intentions were achieved. App users may review this information as well as their answers on the mood survey via the “Personal stats” page and “Journal” on the home screen.

Finally, the “Personal stats” page displays a variety of data collected by the Budd app; for instance, app users can check out their average mood before, after, and 2 days after a chemsex session on monthly and yearly bases. App users can also see how many sessions they have participated in throughout the months (and in total) and for how many hours they were present at a chemsex session on average.

## Discussion

This paper describes the systematic developmental process and ensuing content of an mHealth intervention to support and provide practical tools to chemsex participants to reduce the negative impacts associated with their engagement in chemsex and encourage more reasoned participation.

### Principal Findings

Although using the IMP to develop an intervention involves a time-consuming and complicated process, it proved to be a valuable tool in the planning and development of the Budd app. By conducting the needs assessment, we obtained a comprehensive picture of the health issues. Regularly consulting the external advisory board, performing a literature study, and conducting in-depth interviews made it possible for us to gain insight into, and assign relevance to, the health risks, risk behaviors, and associated behavioral determinants. As a result of the needs assessment and the identification of determinants, the intervention focuses on offering reliable information to users and enables them to self-monitor their behavior, formulate personal intentions, review any discrepancies between these intentions and current behavior, and plan and manage their time. The app components and content were grounded in theory (BCTs) and extensively tested during a pilot study. This resulted in a user-friendly intervention with components that are endorsed by the end users. Steps 5 and 6 of the IMP will be elaborated on in a dedicated article.

Our study has a number of strengths. To our knowledge, this chemsex intervention is the first to use a combination of theoretical grounding and empirical evidence. Second, this process uses a bottom-up approach at all stages by giving potential end users (chemsex participants) and stakeholders an opportunity to co-design the intervention. We involved them throughout all phases of the development process, which allowed the user perspective to play a significant role and increases the likelihood of long-term sustainability of the intervention [33]. Moreover, we have tried to integrate all of the preferences of the potential users as they emerged from the pilot study. Although the intervention was developed in a local context, the lessons learned from using the IMP are generalizable to other settings and contexts, making the lessons relevant to researchers and practitioners internationally, especially given the paucity of evidence-based interventions. This study will provide them with practical and accessible tools and materials that can assist them in setting up health interventions. This knowledge can help other professionals to reduce the amount of time they need to develop their own interventions.

### Limitations

There are some limitations worth noting. Applying all 6 steps of the IMP is a particularly time-consuming task, as has also been mentioned by other studies [104,105]. With complex behaviors such as chemsex participation, identifying the full health problem is a long process. The iterative nature of the IMP has the pitfall of keeping the planning group stuck in the process of exploratory research, pretesting, fine-tuning, and intervention development. It is possible to conduct continual research into determinants for which performance goals are then formulated with accompanying BCTs and PAs. Usually, one does not have the time and resources necessary to carry out the IMP meticulously according to the instructions. In addition, this can become an issue because mHealth interventions evolve rapidly, and creating as well as maintaining an mHealth intervention and ensuring that it stays relevant is critical. We included persons with sufficient experience and expertise on the IMP in the planning group to avoid these issues. In addition, we focused on the personal determinants of unhealthy behavior because the design of the Budd app focuses on the individual user. However, we recognize that social and physical environmental conditions also have a strong impact on behavior [43]. We acknowledge that environmental factors are important in the context of chemsex, such as peer pressure and normalization of drug use (by peers and sexual partners) [11,106,107], a lack of knowledge and expertise among traditional addiction services about chemsex [6,9,26], and experience of shame or stigma regarding sexualized drug use among health care professionals [4,6,8,106,108]. Now that the app is available to the end users, we want to broaden our scope to these possible environmental determinants. As of September 2022, we are working on a follow-up study where we want to look at the health problem from an ecological perspective, as has also been described in the IMP [43]. We aim to explore the interpersonal, organizational, community, and societal factors that influence the behavior of MSM engaging in chemsex. In this way, we can also target the ecological environment of MSM participating in chemsex to attain sustainable behavior change.

### Conclusions

This paper demonstrates how the IMP can be used to design and develop a rigorous theory- and evidence-based intervention to support and inform chemsex participants in reducing the negative impacts associated with chemsex and encourage more reasoned participation. This intervention is unique because no evidence-based mHealth interventions exist yet to support chemsex participants. Notwithstanding the aforementioned limitations, we conclude that our study contributes to the evidence base of chemsex mHealth interventions. The results can be used as input for other mHealth interventions aimed at supporting chemsex participants. The results of the effectiveness study may also contribute to the knowledge about how to support chemsex participants in participating more safely in chemsex sessions.

## Appendix

Multimedia Appendix 1 Milestones of the development process.

Multimedia Appendix 2 Executing tasks in the Budd app.

Multimedia Appendix 3 Post–pilot study interview guide.

Multimedia Appendix 4 Matrices with change objectives.

Multimedia Appendix 5 Theoretical methods and practical applications used in the Budd intervention.

Multimedia Appendix 6 Results of the Budd pilot study.

Multimedia Appendix 7 Overview of the second scope of the Budd app.

## Abbreviations

BCM: behavior change method
BCT: behavior change theory
IMP: intervention mapping protocol
ITM: Institute of Tropical Medicine
mHealth: mobile health
MSM: men who have sex with men
PA: practical application
PrEP: pre-exposure prophylaxis
SCT: social cognitive theory
STI: sexually transmitted infection
SUS: system usability scale
TPB: theory of planned behavior

## Data Availability

The data sets analyzed in this study are available from the corresponding author on reasonable request.
