# Supplementary figures and images for: Using Intervention Mapping to Develop an mHealth Intervention to Support Men Who Have Sex With Men Engaging in Chemsex (Budd): Development and Usability Study
Source: JMIR Res Protoc. 2022 Dec 21;11(12):e39678. doi: 10.2196/39678 (PMC9813820; doi:10.2196/39678)

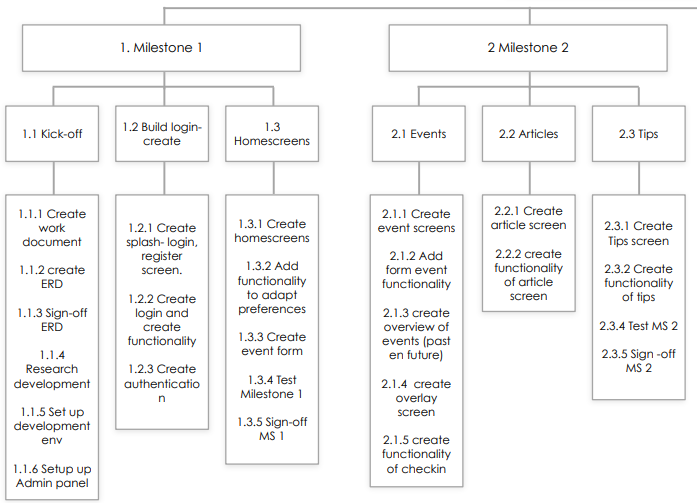


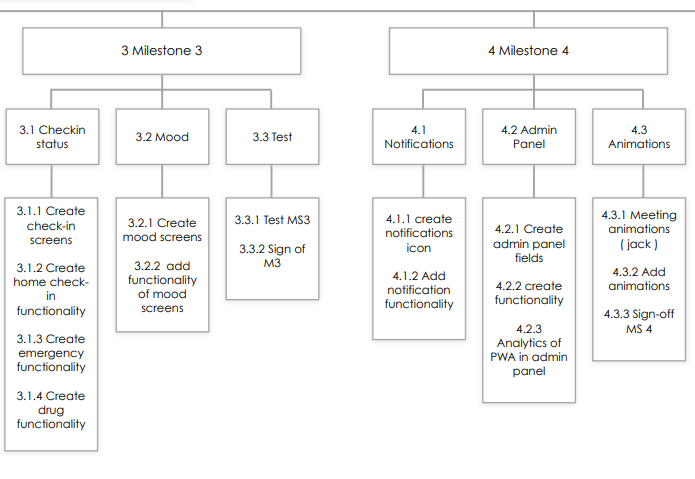


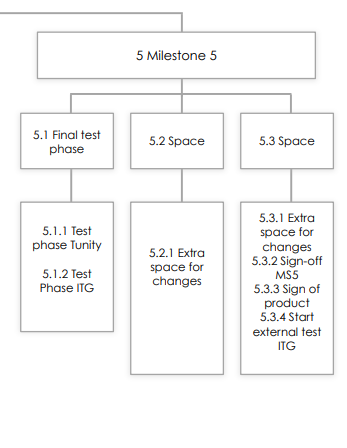

Supplement: Multimedia Appendix 1 [file resprot_v11i12e39678_app1.docx]
