# Supplementary material for: Using Intervention Mapping to Develop an mHealth Intervention to Support Men Who Have Sex With Men Engaging in Chemsex (Budd): Development and Usability Study
Source: JMIR Res Protoc. 2022 Dec 21;11(12):e39678. doi: 10.2196/39678 (PMC9813820; doi:10.2196/39678)
Supplement: Multimedia Appendix 2 [file resprot_v11i12e39678_app2.docx]

Executing tasks in the Budd app

**Information module:**

- Task 1: Create an account (register) & sign in
- Task 2: Add a safety buddy
- Task 3: Look for a drug support service closest to you
- Task 4: Complete your personal checklist (e.g. things you don't want to forget to take to a chemsex session)
- Task 5: Consult the harm reduction tips
- Task 6: Navigate between the different published articles + search for the article concerning STIs

**Dynamic support module:**

- Task 1: Check your calendar to see if you want to schedule an event this month
- Task 2: Add an event (fill in name, date, location and notes)
- Task 3: Import your personal checklist into your checklist for this event
- Task 4: Check in to the event that is currently taking place
- Task 5: Complete the mood survey + notes
- Task 6: Assess the combination of MDMA, GHB and alcohol on safety
- Task 7: Assess what to do if you or someone at the event suffers from overheating
- Task 8: Write something in the notebook
- Task 9: Call your safety buddy
- Task 10: Check-out of the event
- Task 11: Complete the mood survey
