# Supplementary material for: Using Intervention Mapping to Develop an mHealth Intervention to Support Men Who Have Sex With Men Engaging in Chemsex (Budd): Development and Usability Study
Source: JMIR Res Protoc. 2022 Dec 21;11(12):e39678. doi: 10.2196/39678 (PMC9813820; doi:10.2196/39678)
Supplement: Multimedia Appendix 3 [file resprot_v11i12e39678_app3.docx]

# Interview guide post-testing pilot study

1) Design requirements

- What are your thoughts on the design and layout?
  - What do you think of the logo?
  - What do you think of the font?
  - What do you think of the colors used?
  - Is there enough visual variation?
  - Do you think the design fits the theme and target group?
  - Is everything clearly organized?

2) Usability

- How easy or difficult was it to navigate the app?
- What are your thoughts on the language used in the app?
- Was it easy to find the information you were looking for?
- Did you encounter any difficulties as you used the app? Which ones?

3) Features

- What was your favorite feature about the app? Why?
- What was your least favorite feature about the app? Why?
- Are there any features that are missing from the app? Which ones?

4) Acceptability

- How would you describe your overall experience with using the app?
- How many times did you use the app in the past two weeks?
- What surprised you about the app?
- What frustrated you about the app?
- Would you continue to use the app? Why would you not use the app?
- Would you recommend the app to others?
