# Supplementary material for: Using Intervention Mapping to Develop an mHealth Intervention to Support Men Who Have Sex With Men Engaging in Chemsex (Budd): Development and Usability Study
Source: JMIR Res Protoc. 2022 Dec 21;11(12):e39678. doi: 10.2196/39678 (PMC9813820; doi:10.2196/39678)
Supplement: Multimedia Appendix 4 [file resprot_v11i12e39678_app4.pdf]

Matrix 1 – Performance objectives, determinants and change objectives related to **behavioural objective 1 'Chemsex users apply harm reduction measures when taking drugs.'**

| | Determinants | | | | |
|---|---|---|---|---|---|
| Performance objectives | **Awareness** | **Knowledge** | **Self-efficacy** | **Behavioural intention** | **Attitude** |
| *The user avoids dangerous drug combinations.* | - CO1.1 Know which drugs are taken during a chemsex session. | - CO1.2 List which drug interactions need to be avoided and explain why. | - CO1.3 Express confidence in avoiding dangerous drug combinations. | - CO1.4 Plan to avoid dangerous drug combinations. | - CO1.5 Express the necessity to pay attention to the interactions of substances when mixing chemsex drugs. |
| *The user applies dosing-related harm reduction strategies (taking small dosages, leaving sufficient time between dosages).* | - CO1.6 Know the exact doses of drugs taken during a chemsex session.<br>- CO1.7 Know at what times drugs are taken during a chemsex session. | - CO1.8 Recall the correct dosages of most used chemsex drugs.<br>- CO1.9 Describe why it is important to wait sufficiently before redosing.<br>- CO1.10 Describe how long best to wait before taking the next dose of most used chemsex drugs. | - CO1.11 Express confidence in dosing correctly during a chemsex session, despite the effects of the drugs taken and peer pressure.<br>- CO1.12 Feeling capable of waiting long enough before taking the next drug dose. | - CO1.13 Intend to dose correctly during a chemsex session.<br>- CO1.14 Plan to wait sufficiently before redosing. | - CO1.15 Recognise the importance of dosing correctly during a chemsex session.<br>- CO1.16 Recognise the importance of waiting sufficiently before redosing. |
| *The user hydrates and eats sufficiently during the chemsex session.* | - CO1.17 Know the amount of non-alcoholic beverages consumed during a chemsex session.<br>- CO1.18 Know how much food was eaten during a chemsex session. | - CO1.19 Describe why it is necessary to eat and drink sufficiently during a chemsex session.<br>- CO1.20 Describe when and how much non-alcoholic beverages best to drink during a chemsex session.<br>- CO1.21 Describe what best to eat during a chemsex session. | - CO1.22 Express confidence in hydrating regularly during a chemsex session.<br>- CO1.23 Express confidence in eating regularly during a chemsex session. | - CO1.24 Intend to hydrate sufficiently during a chemsex session.<br>- CO1.25 Intend to eat sufficiently during a chemsex session. | - CO1.26 Express the importance of hydrating regularly during a chemsex session.<br>- CO1.27 Express the importance of eating regularly during a chemsex session. |
| *The user brings harm reduction materials to the chemsex session (e.g. drinking straw, snuff tube, clean needles, own (tested)drugs).* | - N/A | - CO1.28 List the harm reduction materials that need to be brought to a chemsex session. | - CO1. 29 Being sure to bring harm reduction materials to a chemsex session. | - CO1.30 Plan to bring necessary harm reduction materials to a chemsex session. | - CO1.31 State the importance of bringing harm reduction materials to a chemsex session. |

Matrix 2 – Performance objectives, determinants and change objectives related to **behavioural objective 2 'Chemsex users deliberately plan their participation in chemsex parties.'**

| | Determinants | | | | |
|---|---|---|---|---|---|
| Performance objectives | **Awareness** | **Knowledge** | **Self-efficacy** | **Behavioural intention** | **Attitude** |
| *The user prepares for participation in a chemsex session.* | - CO2.1 Aware of preparatory measures taken. | - CO2.2 Explain how to best prepare for a chemsex party (setting a curfew, plan to get home safely, setting personal limits, set reminders, discuss sexual preferences). | - CO2.3 Express confidence in taking preparatory measures before participating in a chemsex session. | - CO2.4 Plan to prepare for participation in a chemsex session. | - CO2.5 Recognize the importance of preparing for participation in a chemsex session. |
| *The user plans sufficient time after a chemsex session to recover mentally and physically.* | - CO2.6 Aware of time needed after a chemsex session to recover mentally and physically. | - CO2.7 Describe possible impact on mental and physical well-being after participating in a chemsex event.<br>- CO2.8 Explain how to best tackle the comedown. | - CO2.9 Express confidence in scheduling enough time after a chemsex session to recover mentally and physically. | - CO2.10 Plan to recover as well as possible the days following participation in chemsex. | - CO2.11 Recognize importance of taking care of body and mind, and give them a rest the days after participating in chemsex. |

Matrix 3 – Performance objectives, determinants and change objectives related to **behavioural objective 3 'Chemsex users take the step towards support services when needed.'**

| | Determinants | | | | |
|---|---|---|---|---|---|
| Performance objectives | **Awareness** | **Knowledge** | **Self-efficacy** | **Behavioural intention** | **Attitude** |
| *The user takes the step to assistance and support in time.* | - CO3.1 Aware of own participation in chemsex (frequency and duration).<br>- CO3.2 Aware of fluctuations in mood/mental health. | - CO3.3 List where to go for psychological support for chemsex related questions or support in Flanders.<br>- CO3.4 List where to go for peer support.<br>- CO3.5 Recall where to go to get drugs tested.<br>- CO3.6 List HIV/AIDS reference centres in the area.<br>- CO3.7 Describe which signs may indicate problematic chemsex use. | - CO3.8 Express confidence in seeking appropriate assistance when needed. | - CO3.9 Plan to seek chemsex support when needed. | - CO3.10 Find it useful to seek chemsex support when needed. |

Matrix 4 – Performance objectives, determinants and change objectives related to **behavioural objective 4 'Chemsex users adhere to their HIV medication or PrEP (if applicable)'**

| | Determinants | | | | |
|---|---|---|---|---|---|
| Performance objectives | **Awareness** | **Knowledge** | **Self-efficacy** | **Behavioural intention** | **Attitude** |
| *The user takes HIV medication or PrEP timely during the chemsex session.* | - CO4.1 Aware of HIV medication/PrEP intake during a chemsex session. | - CO4.2 Explain why it is necessary to take HIV medication/PrEP on time. | - CO4.3 Express confidence in taking HIV medication/PrEP correctly during a chemsex session. | - CO4.4 Plan to take HIV medication/PrEP correctly when participating in chemsex. | - CO4.5 Recognise the importance of taking HIV medication/PrEP correctly. |

Matrix 5 – Performance objectives, determinants and change objectives related to **behavioural objective 5 'Chemsex users assist others during a chemsex party.'**

| | Determinants | | | | |
|---|---|---|---|---|---|
| Performance objectives | **Awareness** | **Knowledge** | **Self-efficacy** | **Behavioural intention** | **Attitude** |
| *The user contacts the emergency services in case of an emergency.* | - CO5.1 Recognize when others are in an emergency situation. | - CO5.2 List symptoms that indicate a need for emergency help.<br>- CO5.3 Explain how to best contact emergency services. | - CO5.4 Express confidence in contacting 112 in case of an emergency during a chemsex session. | - CO5.5 Plan to contact emergency services in case of an emergency during a chemsex session. | - CO5.6 Recognize the importance of emergency services and their help in case of emergencies during a chemsex session. |
| *The user gives first aid to others.* | - N/A | - CO5.7 List possible situations during a chemsex session where others would need support (overheating, paranoia, feeling nauseous, etc.).<br>- CO5.8 Explain how best to help others in those situations. | - CO5.9 Express confidence in helping others at a chemsex session when they feel unwell. | - CO5.10 Intend to help others in case of need. | - CO5.11 Recognize importance of helping others at a chemsex session when they feel unwell. |
