# Supplementary material for: Using Intervention Mapping to Develop an mHealth Intervention to Support Men Who Have Sex With Men Engaging in Chemsex (Budd): Development and Usability Study
Source: JMIR Res Protoc. 2022 Dec 21;11(12):e39678. doi: 10.2196/39678 (PMC9813820; doi:10.2196/39678)
Supplement: Multimedia Appendix 5 [file resprot_v11i12e39678_app5.pdf]

Theoretical methods and practical applications used in the Budd intervention

| Behaviour change method (BCM) | Definition | Determinant | Practical application (PA) |
|---|---|---|---|
| **BCM1**: Providing information on:<br>- chemsex-related risks;<br>- health consequences;<br>- when and where to perform health promoting behaviour;<br>- how to perform health promoting behaviour;<br>- emotional consequences | General information about risk behaviour, benefits and costs of action/inaction, advice on how to perform a behaviour, health consequences. | Knowledge, attitude, behavioural intention | - **PA1**: Drug information (CO1.8, CO1.9, CO1.10, CO1.15)<br>*Common used chemsex drugs with short info on dosage, duration, effects*<br>- **PA2:** Drug combination tool (CO1.2, CO1.5)<br>*A tool to assess the interaction of most used chemsex drugs*<br>- **PA3**: Articles about chemsex-related topics (CO1.9, CO1.19, CO1.20, CO1.21, CO1.28, CO4.2, CO4.5, CO2.2, CO2.7, CO2.8, CO3.7, CO5.3, CO5.7, CO5.8, CO1.16, CO1.26, CO2.5, CO1.26, CO1.27, CO1.31, CO2.5, CO5.6, CO5.1)<br>- **PA4**: Overview of healthcare and support in Flanders (CO3.3, CO3.4, CO3.5, CO3.6)<br>- **PA5**: Emergency information (CO5.2, CO5.7, CO5.8, CO1.26, CO5.11, CO5.5, CO5.1)<br>*How best to act in a range of chemsex-related emergency situations + quick button to call emergency services*<br>- **PA6**: Testimonials from other GBMSM who participate in chemsex (CO2.7, CO2.8, CO3.7, CO2.11, CO3.10, CO5.10)<br>*Other users' experiences on peer pressure, comedown, reasons for using Budd, and the progression from recreational to more problematic use*<br>- **PA7**: Chemsex knowledge quiz (CO1.2, CO1.8, CO1.10, CO1.20, CO1.21, CO1.28, CO4.2, CO5.2, CO5.8)<br>*Multiple-choice questions about the content of the app, with feedback per completed question* |
| **BCM2**: Self-monitoring of behaviour | Prompting the person to keep a record of specified behavior(s). | Awareness, knowledge, self-efficacy | - **PA8**: Mood survey (CO2.6, CO3.2)<br>*The question 'How are you doing' is asked in relation to chemsex sessions: at check-in, check-out, and 2 days later during comedown*<br>- **PA9**: Notebook with timestamps during chemsex-session (CO1.1, CO1.6, CO1.7, CO1.17, CO1.18, CO4.1)<br>(e.g. for monitoring of drug intake, food intake)<br>- **PA10:** Journal (CO3.2, CO3.9)<br>*Reported moods, notebook entries and reflections on preparation tool are saved in a journal. User can also make entries in journal.*<br>- **PA11**: Personal checklist specific to a chemsex session (CO4.4, CO2.5, CO2.4, CO1.29, CO2.3)<br>*List of harm reduction materials to bring to a chemsex session.*<br>- **PA12**: Reflection on preparation tool (CO1.1, CO2.1, CO2.6, CO4.1)<br>- **PA13**: Personal statistics (CO2.6, CO3.1, CO3.2)<br>*Visual representation of mood, number of chemsex sessions, evolution of chemsex sessions per month, many hours one spends on average at a chemsex date/party.* |
| **BCM3**: Goal setting | Encourage the user to decide to act or set a general goal. | Behavioural intention, self-efficacy, attitude | - **PA14**: Preparation tool (CO1.4, CO1.13, CO1.14, CO 4.3, CO4.4, CO1.31, CO2.5, CO2.11, CO1.30, CO2.4, CO2.10, CO1.3, CO1.29, CO2.3, CO2.9, CO1.11, CO1.22, CO1.1.23, CO1.24, CO1.25)<br>*A tool with a series of questions to prompt the user to formulate intentions related to a chemsex session*<br>- **PA15**: Personal checklist (CO4.4, CO2.5, CO2.4, CO1.29, CO2.3) |

| | | | |
|---|---|---|---|
| **BCM4**: Review discrepancy between current behaviour(s) and intention(s) | Draw attention to discrepancies between the users' current behaviour and the person's previously set outcome goals, behavioural goals or action plans. | Awareness, behavioural intention, self-efficacy | - **PA12** Reflection on preparation tool (CO2.1, CO2.6, CO2.9, CO3.8, CO1.11, CO1.12)<br>*Budd notifies user two days after the chemsex session to reflect on set intentions and actual behaviour* |
| **BCM5**: Planning/time management | Helping the person consciously plan the behaviour. | Behavioural intention, self-efficacy | - **PA16**: Planning tool chemsex session (CO2.4, CO2.10, CO2.9)<br>*Schedule a future chemsex session in the app using pre-set questions* |
| **BCM6**: Social support (unspecified) | Advise on, arrange or provide social support (e.g. from friends, relatives, colleagues,' buddies') for performance of the behaviour. It includes encouragement and counselling. | Behavioural intention | - **PA17**: Advise the user to call a 'safety buddy' when they need help or support during a chemsex session (CO3.9, CO5.10) |
| **BCM7**: Credible source | Present verbal or visual communication from a credible source in favour of or against the behaviour | Attitude | - **PA18**: Make it clear that the app comes from a reliable source with expertise on the topic<br>*(Institute of Tropical Medicine and members of the planning group)* |
