# Supplementary material for: Using Intervention Mapping to Develop an mHealth Intervention to Support Men Who Have Sex With Men Engaging in Chemsex (Budd): Development and Usability Study
Source: JMIR Res Protoc. 2022 Dec 21;11(12):e39678. doi: 10.2196/39678 (PMC9813820; doi:10.2196/39678)
Supplement: Multimedia Appendix 6 [file resprot_v11i12e39678_app6.pdf]

CHEMIFIED:

results pilot study

May 2021

INSTITUTE OF TROPICAL MEDICINE ANTWERP

Overview

- Pilot study
  - Overview participants
  - Performing tasks (usability issues)
  - System Usability Scales
  - Follow-up interview

Overview participants

	age (years)	education	employment status	frequency (chemsex)	mobile phone usage (per day)	use of mobile health applications
1	40-49	Diploma secondary school	Disabled	less than once a month	more than 3h	No
2	50-59	Master	Full-time	weekly	1.5 to 2h	Yes
3	30-39	Diploma secondary school	Unemployed	monthly	1.5 to 2h	No
4	older than 60	Diploma secondary school	Retired	weekly	30min to 1h	Yes
5	30-39	No secondary school diploma	Unemployed	weekly	1.5 to 2h	No
6	older than 60	Professionel bachelor	Full-time	weekly	30min to 1h	No
7	30-39	PhD	Full-time	monthly	2,5u tot 3u	No
8	25-29	Master	Full-time	monthly	more than 3h	Yes

USABILITY ISSUES

Overview of healthcare services

Task: Look for a drug support service closest to you

- Not efficient that you have to copy paste the e-mail address, mobile phone number and address provided

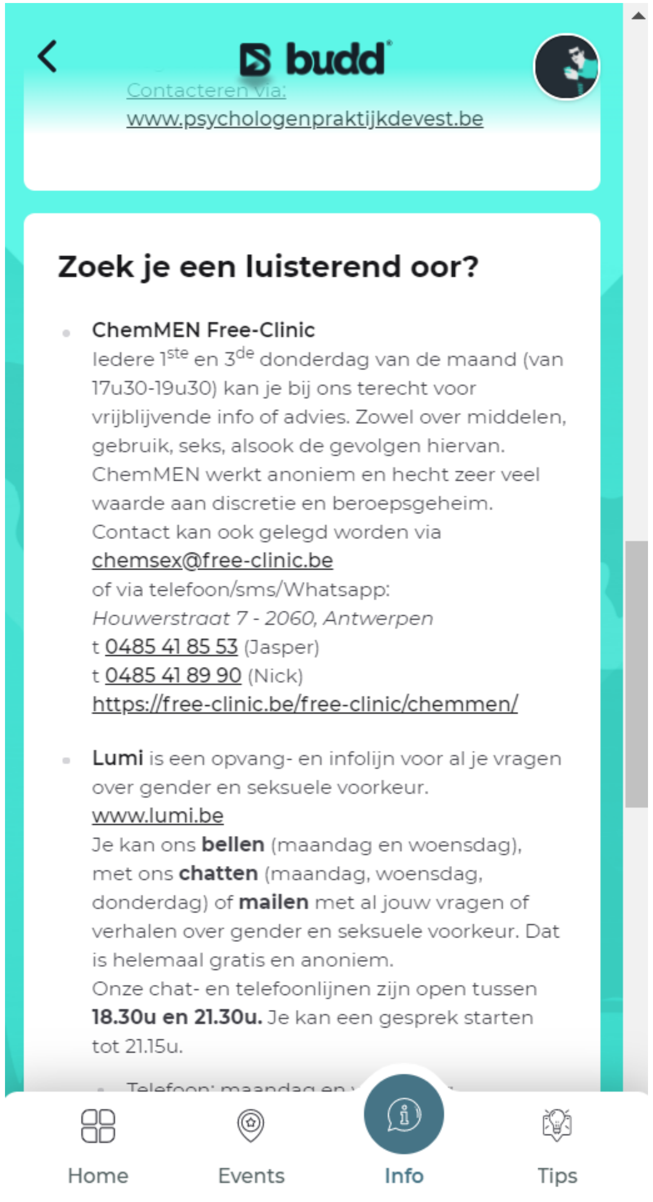

Articles

Task: Navigate between the different published articles + search for the article concerning STIs

- Not every article starts with an introduction
- It is time consuming that you can not search the articles based on key words → add search function

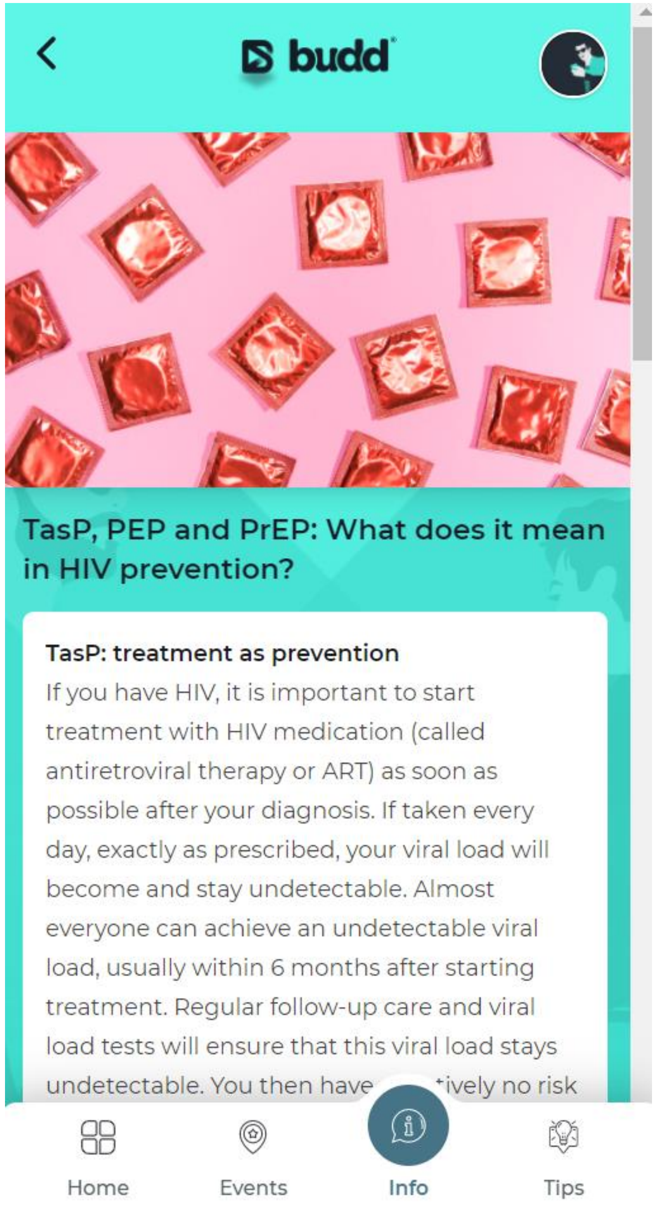

Safety buddy

Task: Add a safety buddy

- Input field is unclear: the example telephone number seems real
- Time consuming that you have to copy paste from your contacts → Add functionality to add buddy from your contacts?
- Unclear that avatar is clickable

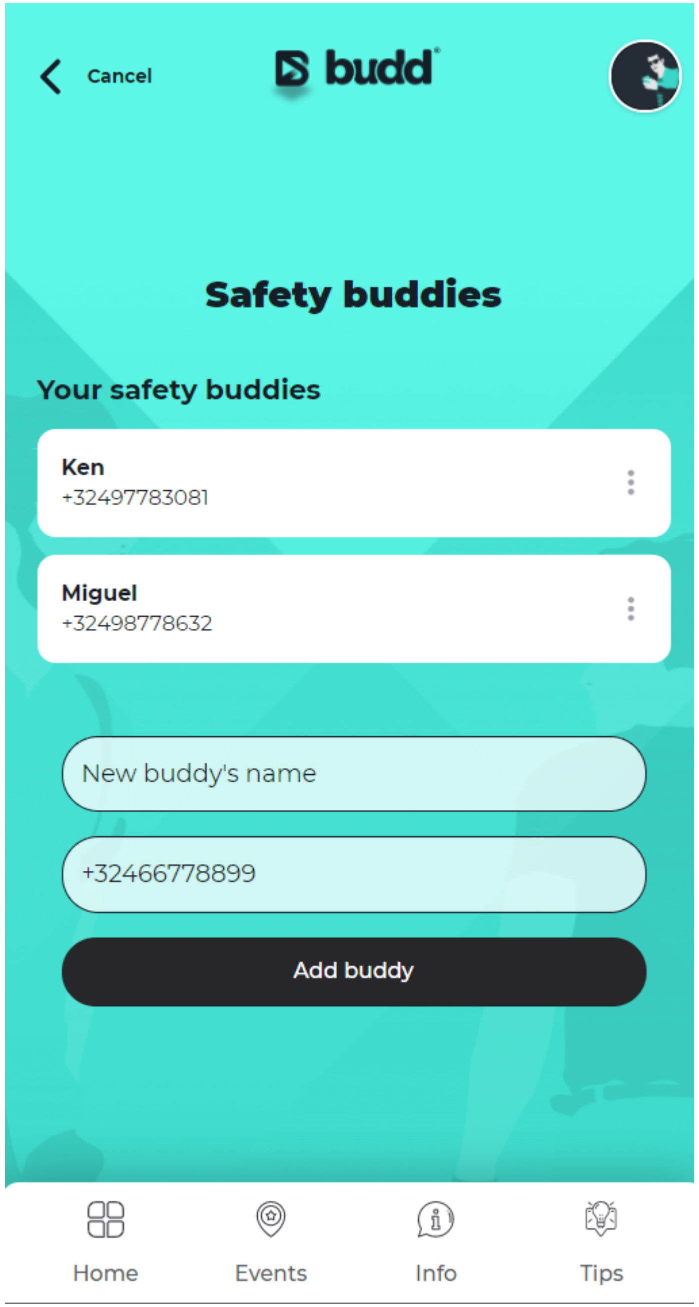

Checklist

Task: Complete your personal checklist (e.g. things you don't want to forget to take to a chemsex session)

- Is not logical under ‘info menu’

→Move to personal settings/events?

- Unhandy to use because too many steps to delete an item → add functionality delete by sliding?
- ‘add item’ at the top, with safety buddy at the bottom
- Not clear that the ‘arrow to go back’ belongs to the pop-up
- Needs more explanation: what is the purpose of using the checklist, and how can you best use it?
- Event-specific checklist: 'let Budd suggest', if you choose 'personal items' you cannot return again

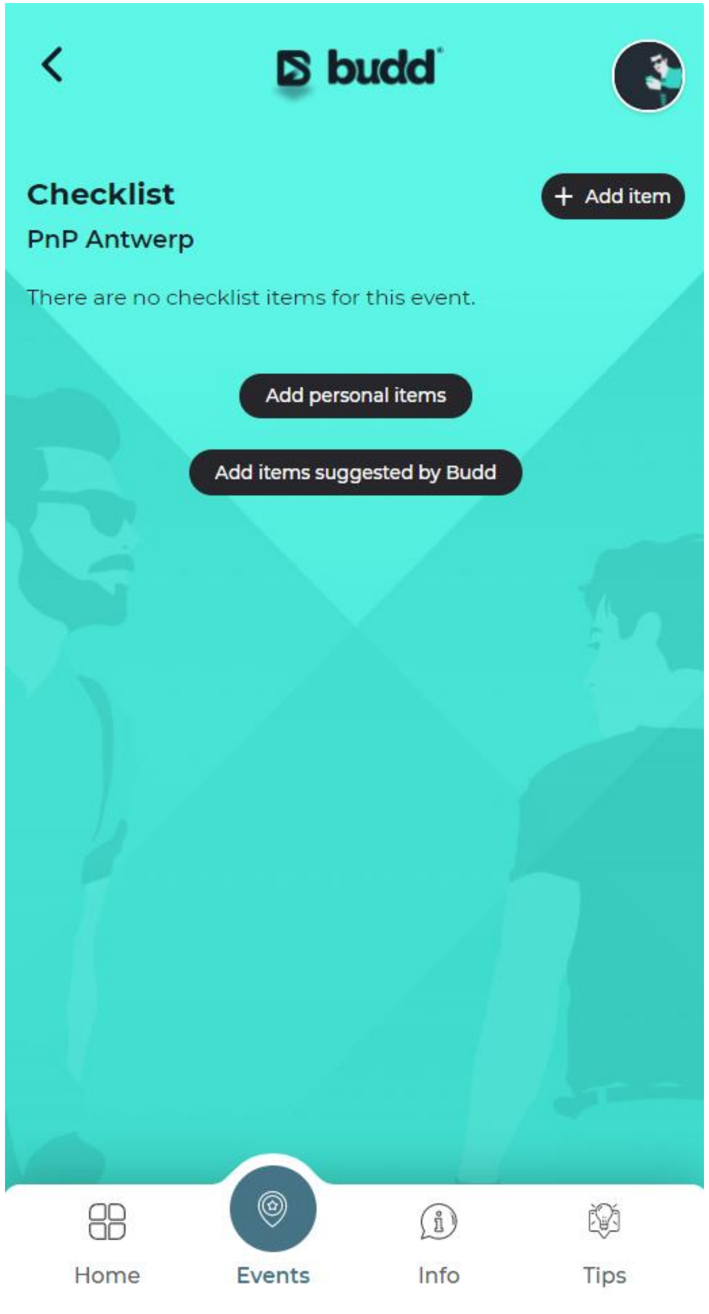

Tips

Task: Consult the harm reduction tips

- Is placed too central → integrate in other tool?

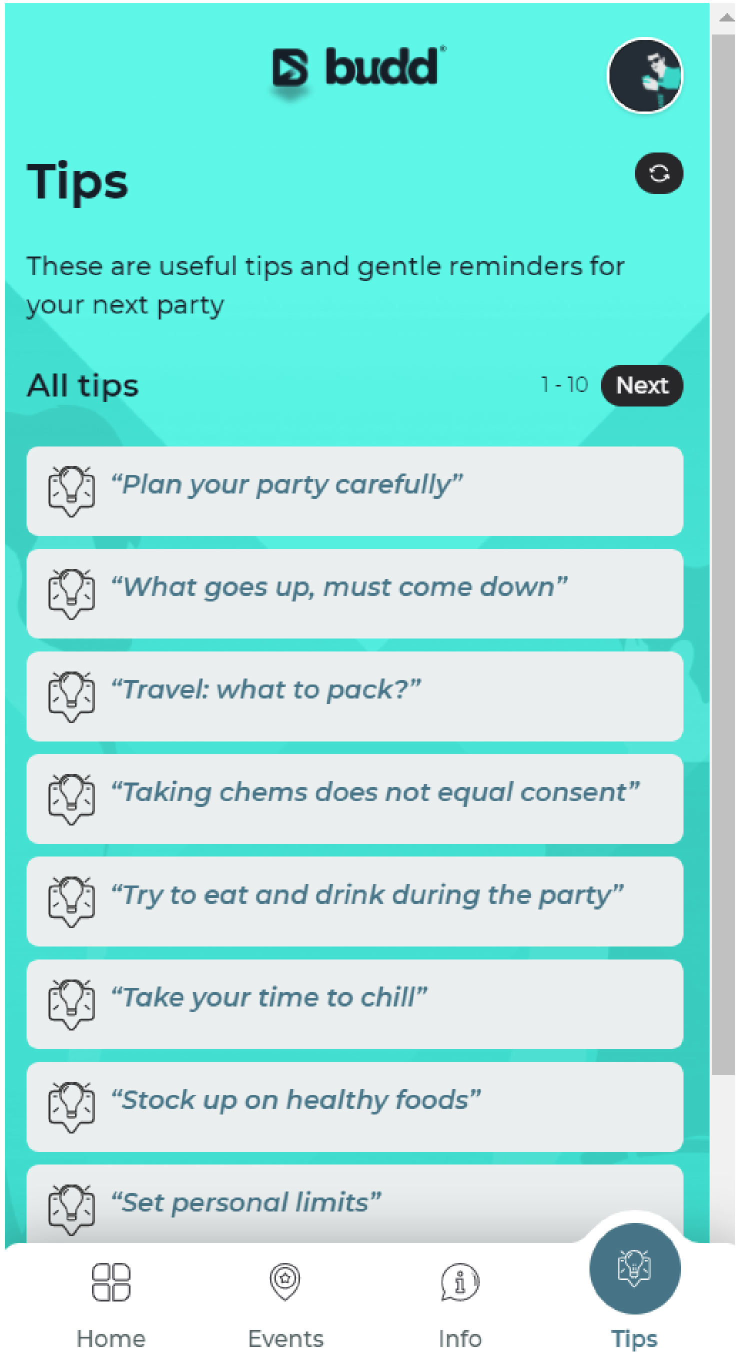

Add event

Task: Add an event (fill in name, date, location and notes)

- Add starting time to event
- Would be easy to link Google Maps to location for the directions
- Too many steps/clicks to delete a created event → delete created events by swiping

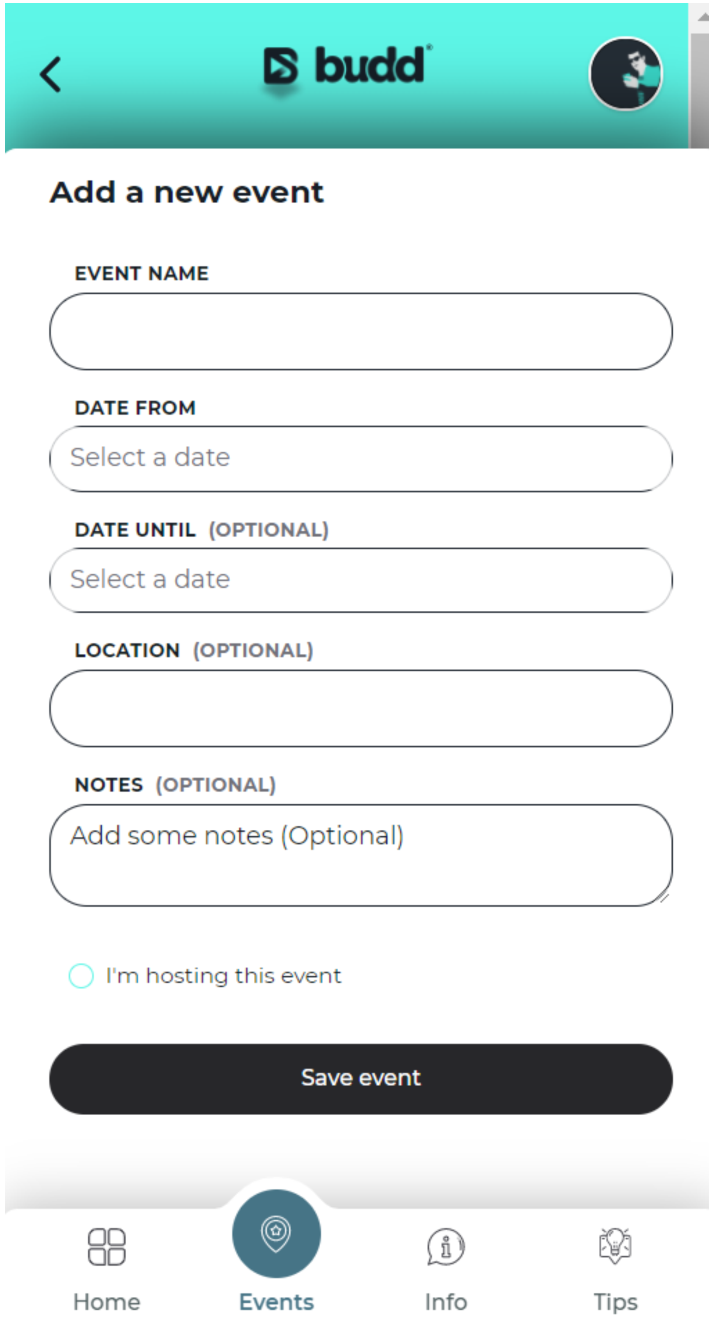

Check-in

Taak: Check in to the event that is currently taking place

- Unclear that you have to slid to check in. The checkmark is confusing.
- It is annoying that you can not get back to other app content during event.

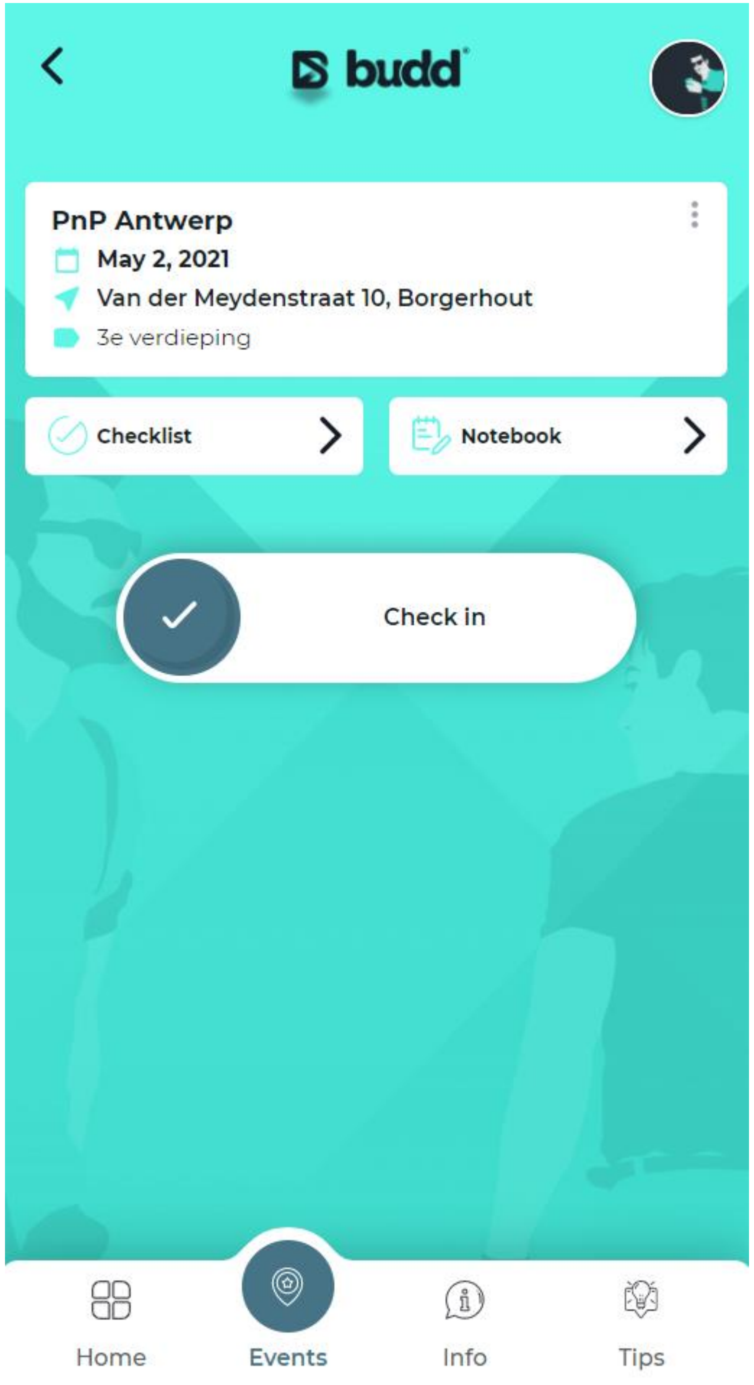

Mood survey

Task: Complete the mood survey

- Not clear that you can move the dot
- Difference between moods is unclear
  - Maybe add fase with wink and tongue: used to mean ‘horny’ and also often used via dating apps in chats

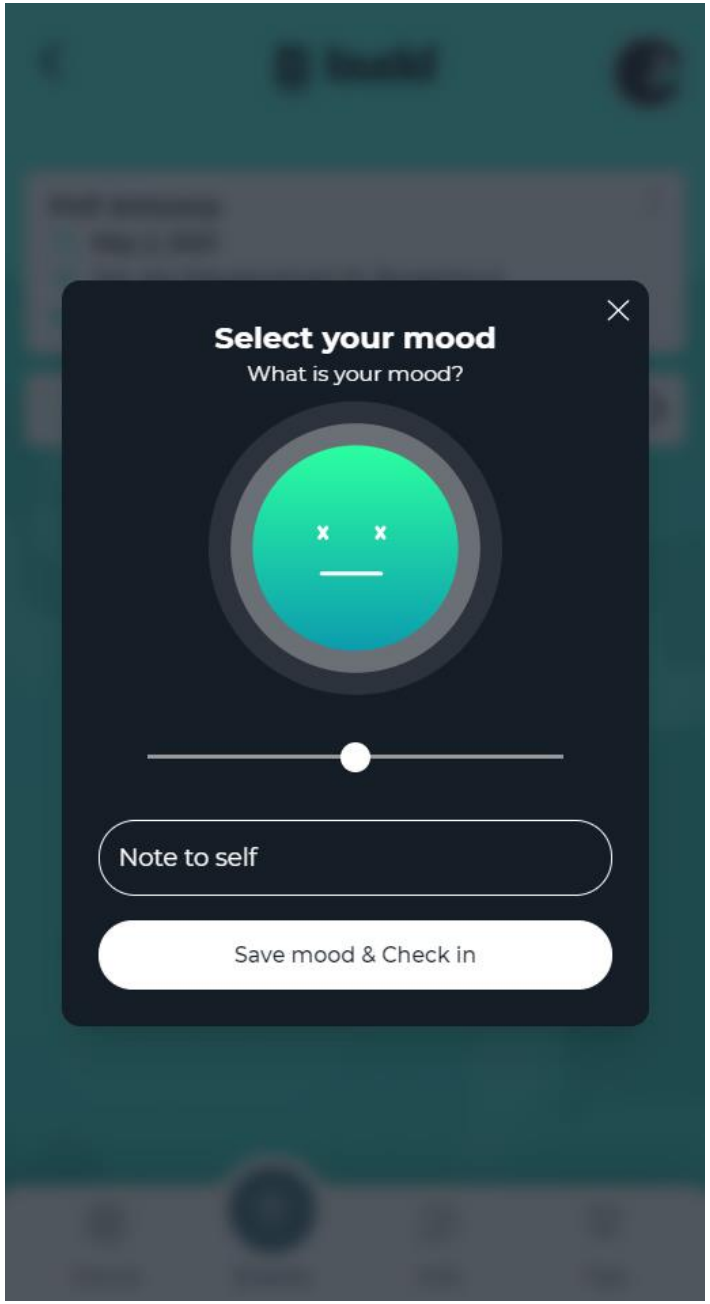

Drug combination tool

Task: Assess the combination of MDMA, GHB and alcohol on safety

- Only word ‘drug co’ visible on iPhone SE
- ‘Deselect’ is not clear → Add cross?
- Not clear that you can enter multiple combinations, there is only one dropdown menu initially
- Already add what can go wrong and how best to act in such cases

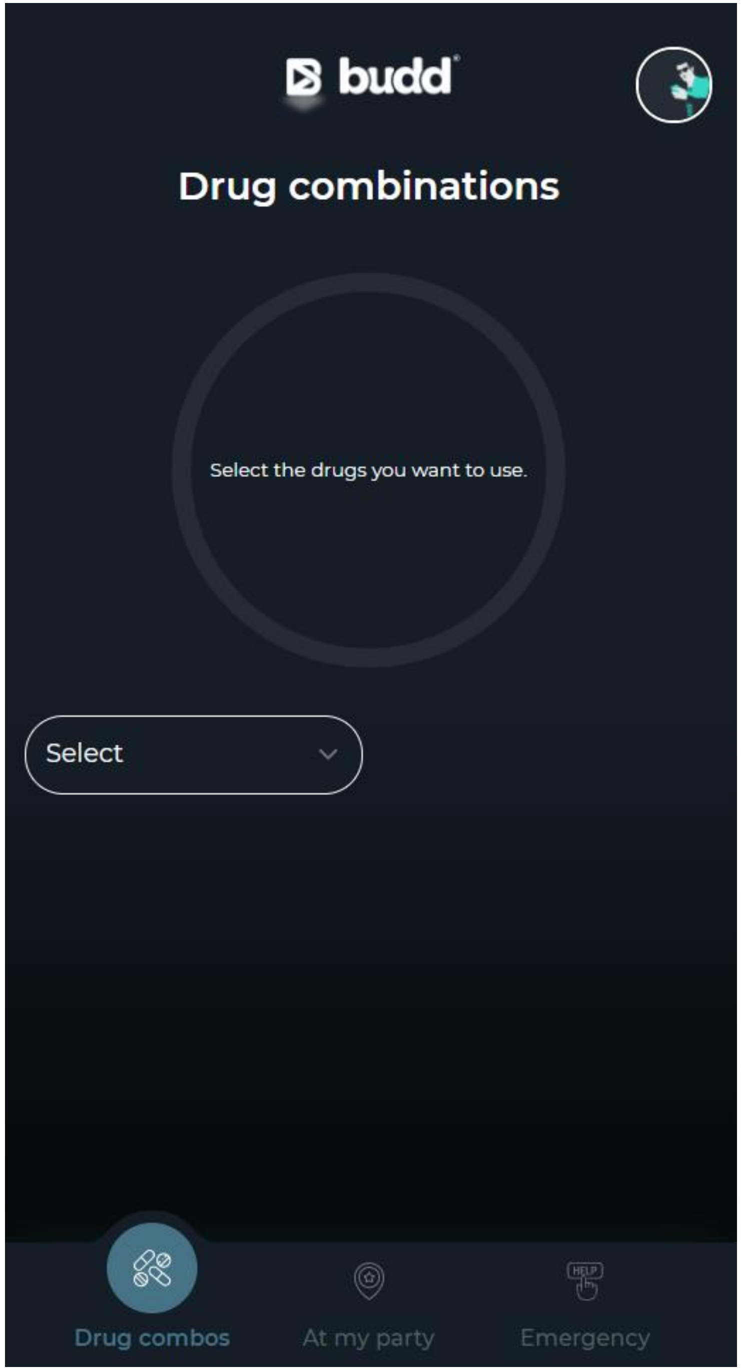

Emergency info

Task: Assess what to do if you or someone at the event suffers from overheating

- Red bar 'call emergency services' overlaps text at bottom, not all readable
- Paragraph is too long, especially when you have taken chems → Less text and clearer action points.

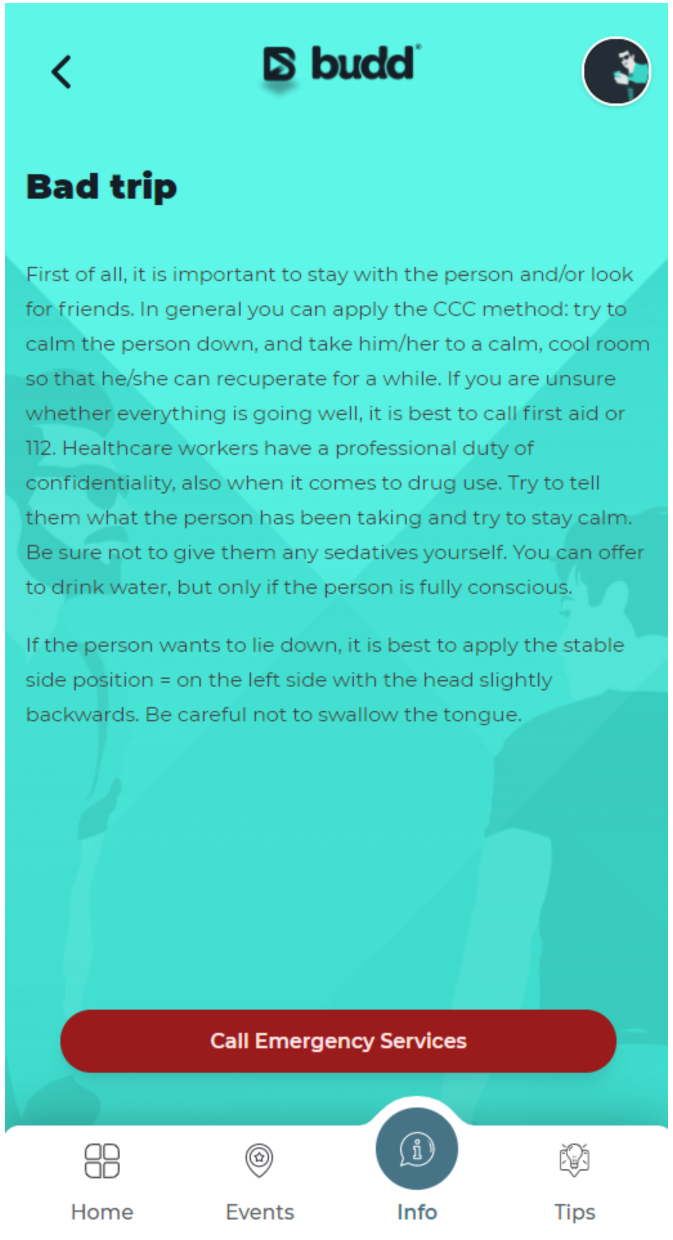

Notebook

Task: Write something in the notebook

- Emoticons are too small, facial expressions are difficult to read
- Minimum of three characters is not convenient. Sometimes working with abbreviations to avoid writing drugs in full
- Not possible to delete notes → Add function: delete note

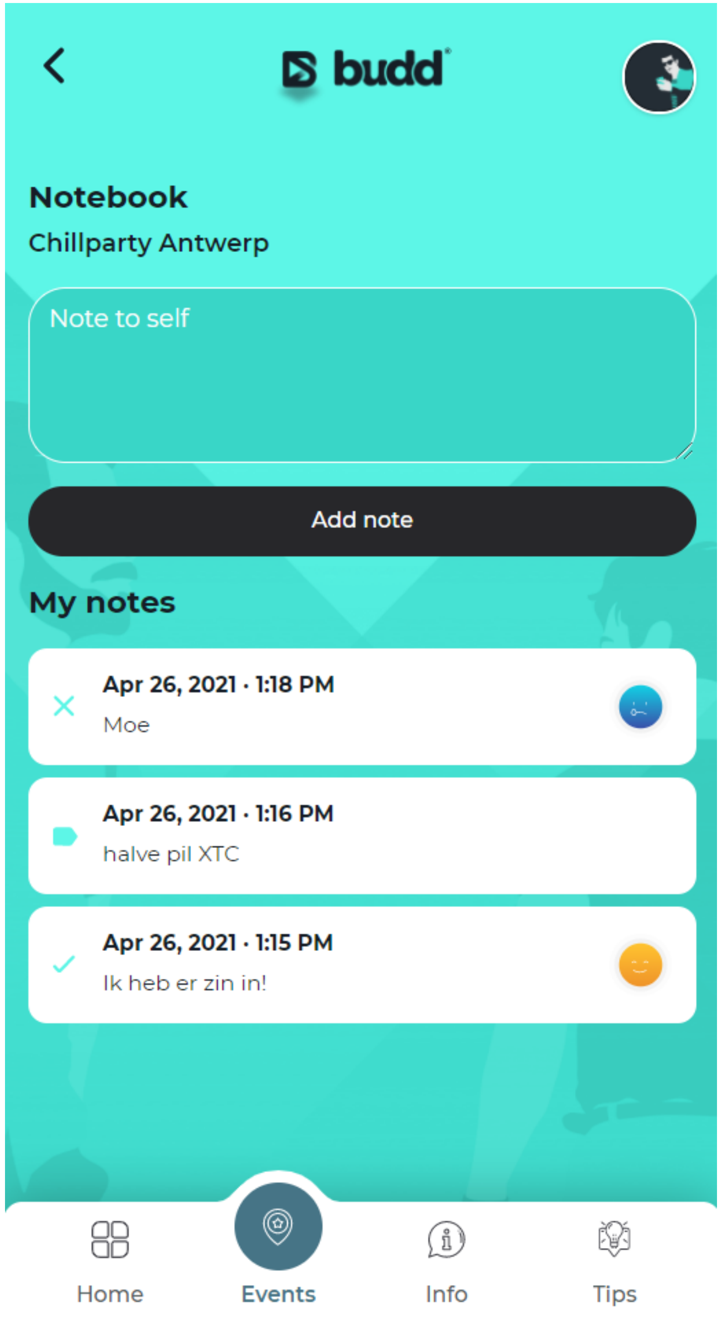

Safety buddy - calling

Task: Call your safety buddy

- Put button in multiple places

→ ‘emergency’

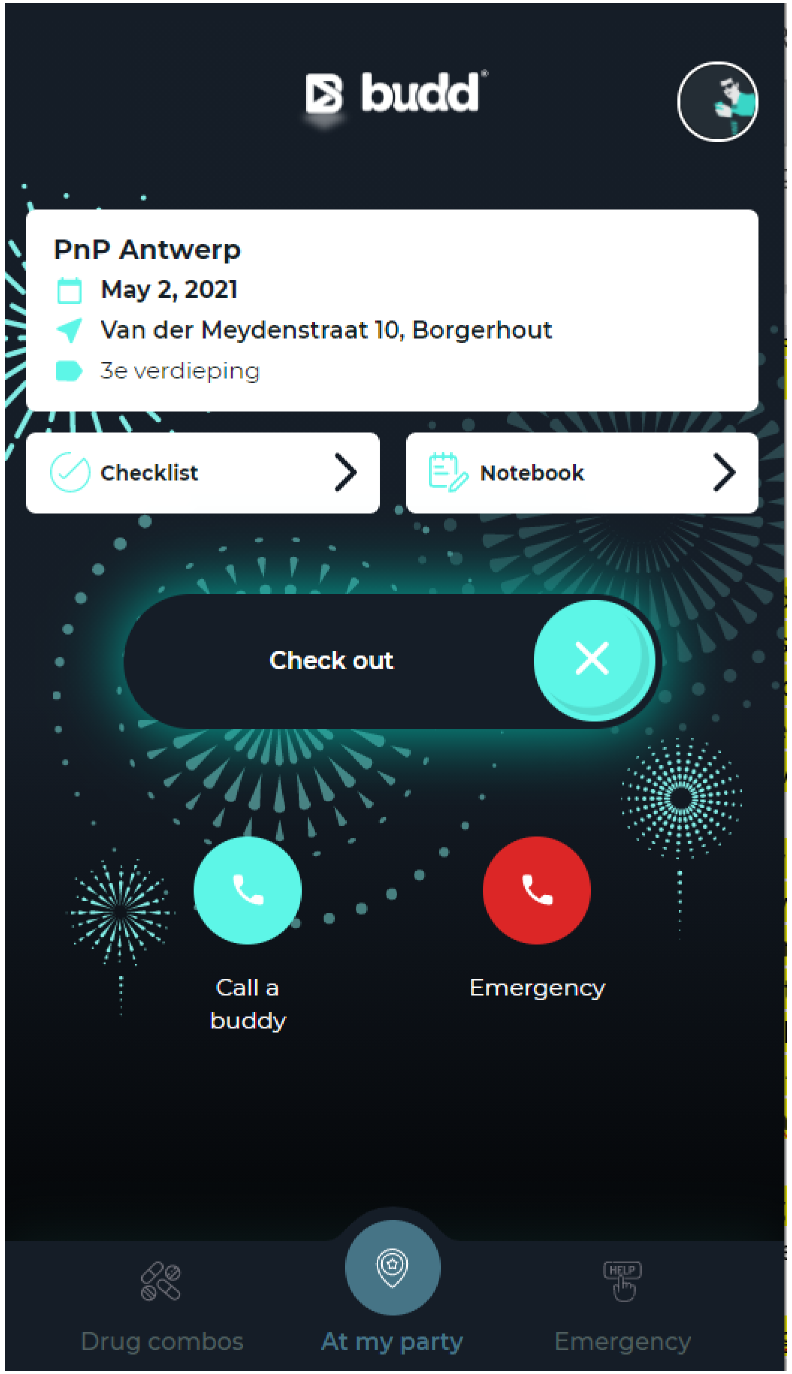

Calendar

Task: Check your calendar to see if you want to schedule an event this month

- Holidays are currently not yet visually marked in the calendar, which is sometimes annoying when trying to schedule chemparties

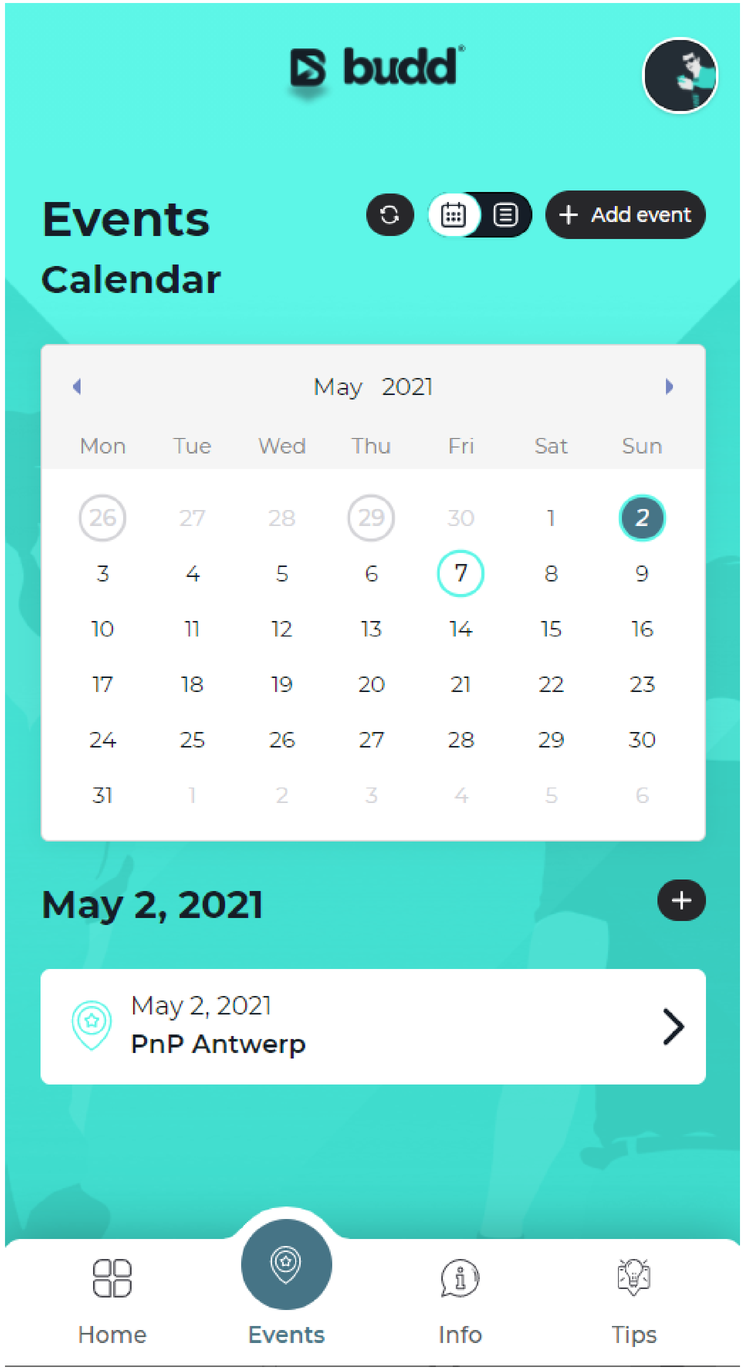

SYSTEM USABILITY SCALE

Results - SUS

- participant 1: 62,5
- participant 2: 80
- participant 3: 87,5
- participant 4: 87,5
- participant 5: 65
- participant 6: 80
- participant 7: 77,5
- participant 8: 75

→ **Mean: 76,88**

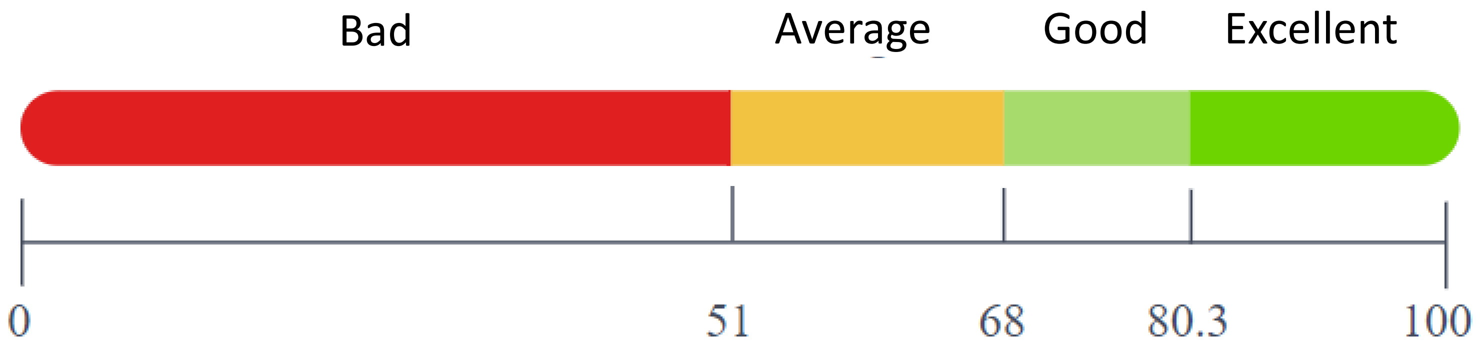

FOLLOW-UP INTERVIEW

Overview

1. Design requirements
    - Logo, font, colours, design
2. Usability
    - Navigation within app
    - Language
3. Features
    - Favourite/least favourite
    - What is missing?
4. Acceptability
    - Overall experience (strengths + areas for improvement)
    - Frequency of use
    - Would you continue to use the app?
    - Would you recommend it to others?

1. Design requirements

1. Layout
   - Layout/structure is clear
   - Could be more consistent in terms of user interface
     - Pop-up, arrow on underlying screen
   - ‘At my party' can be confusing, add an easy way to get to the general section
   - Put next event on home screen if no event is planned on that day itself
   - Personal checklist belongs in 'settings’
   - Put tips under 'info' or 'settings' ('my saved tips')

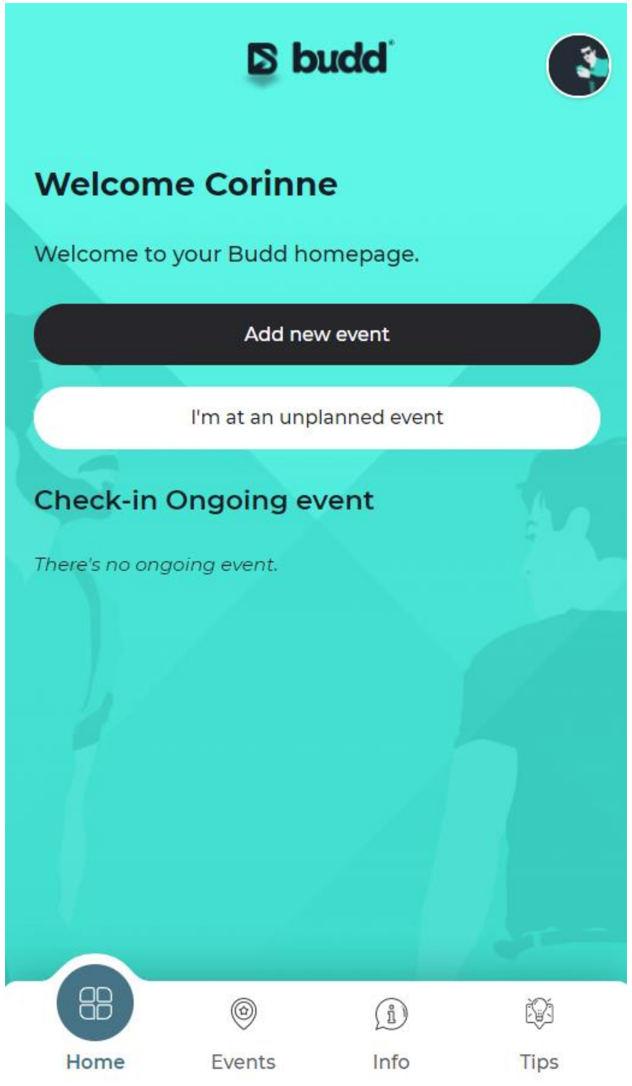

2. Font

(no comments)

- Easy to read
- Clear, simple

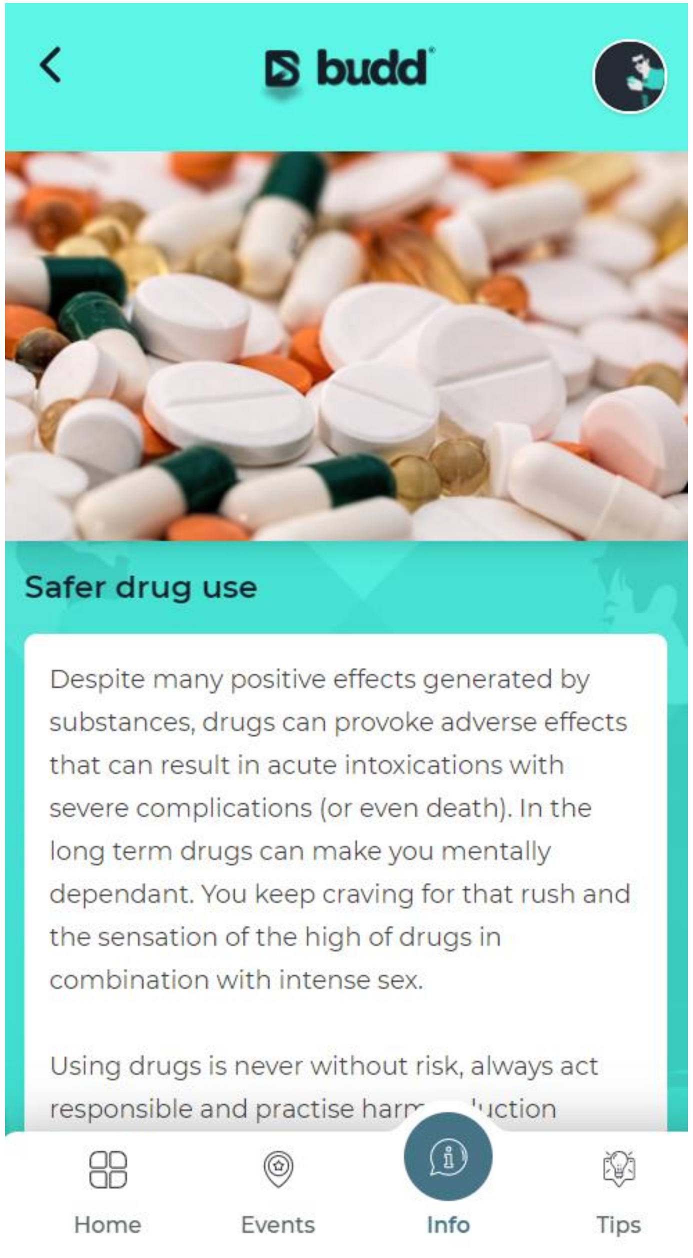

3. <u>Colours</u>
   - Neutral
   - Clear distinction dark display during event - other part of the app
   - Green comes across as 'clinical'
   - Calm
   - Feeling 'we take care of you'

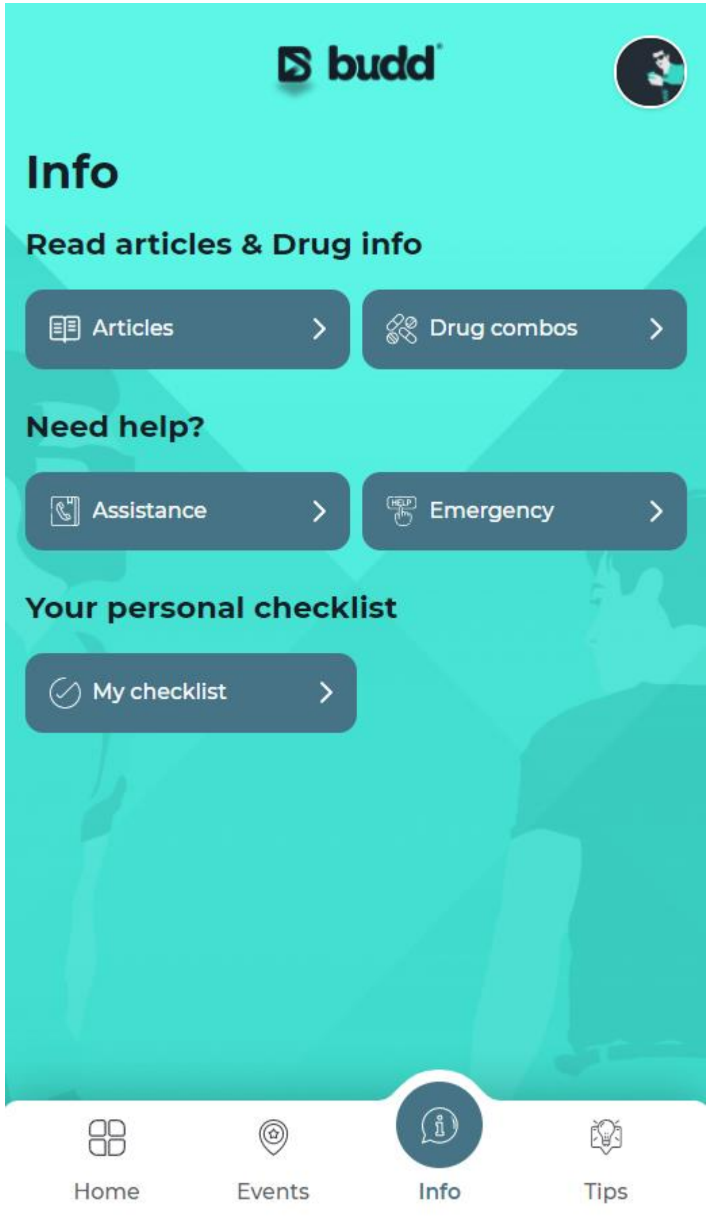

2. Usability

1. <u>Navigation within app</u>
   - Easy (7)
   - During event no longer clear what notebook was for
   - Confusing that you're in another section during the event and can't go back without checking out
   - Less user-friendly that you can only view events separately per event. Impractical to compare → needs an overview

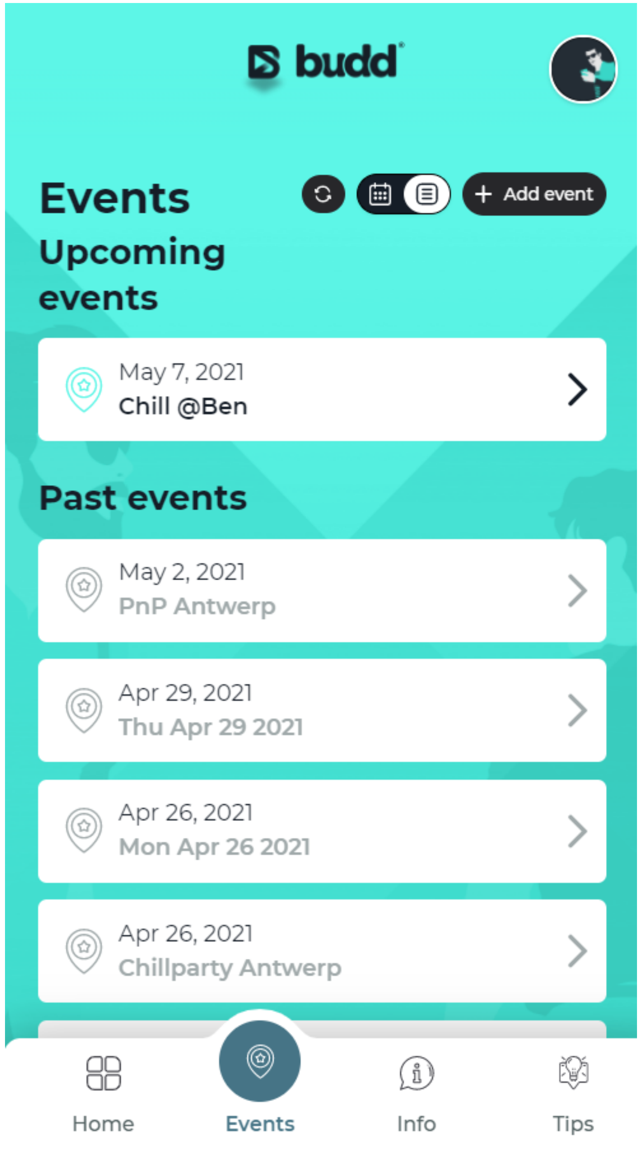

2. <u>Language</u>

- Replace term 'drugs' with 'chems' (6)
  - More familiar
  - Less heavy
  - In touch with key population
- Tailor word choice more to the user (3)
  - Sometimes too academic/difficult
  - Not clear enough for general audience (especially under the influence of substances)

3. <u>Finding information</u>
    - Tips are in a less logical place
    - In articles, you cannot search by words

3. Features | *favorite features*

- Information module + articles (4)
- Drug combinations (3)
- Personal checklist (2)
- Notebook (3)
- Buddy (2)
  - Further development needed (extra options)

3. Features | least favourite ***feature***

- Scheduling and monitoring events (2)
  - Because of limited frequency chemsex participation
  - Mainly unplanned (‘impulsive’) chemsex participation
- Articles (3)
  - Too little elaborated (information about most used chemsex substances is needed)
  - Users feel they already have sufficient chemsex knowledge (2)
- Personal checklist (2)
  - Experienced user, less relevant (2)

Recommendations (What is missing?)

- <u>Other users</u>
  - Ability to interact with other users
  - Forum over which ITM has control: add short experiences/testimonials from other chemsex participants
  - Enable adding friends
    - People you meet at an event
    - Establish some kind of connection with other people via the app

Recommendations (What is missing?)

- <u>Reminders</u>
  - Sometimes forgets to check out of the event when going home
    - Reminder: 'don't forget to check out'
  - Forgets to use the app
    - Push notification: reminder that users can start an unplanned event within the app
  - Checklist
    - Before the event: 'don't forget to check off your personal checklist'

- <u>Contact tracing</u>
  - Anonymously store with whom you have been in contact with. If an STI is detected, other attendees can be notified anonymously via Budd.

Recommendations (What is missing?)

- <u>Overview across events</u>
  - Ability to view all collected data in summary form (averages, evolution)
    - Combersome to consult per event currently
    - Would also be useful to be able to discuss with healthcare professional for follow-up in an assistance programme
- <u>General drug info</u>
  - Per drug: What is the effect? What are the possible dangers? How do I recognise it?
- <u>Dosages/usage quantities</u>
  - Would provide guidance

4. Acceptability

1. Frequency of use
    - Used 2 times
    - Just before a date (in preparation) and during (2)
    - 10 x used
    - A few times, “Oh yes I am participating in this study, I want to use this correctly”
    - Once during a date and a few times before and after
    - Used 3 times a week
    - A few times to go through all the information. Not used for dates.

2. Would you continue to use the app?
   - Yes, for the informative part
   - Yes, but it is important to think about how we will motivate others
   - Yes, because I am dutiful
   - I would definitely download and use it, but not always. Depends on how often I participate in events.
   - Yes, because it has a scientific basis
   - Yes, to monitor my usage
   - Yes, for entering my doses
   - Yes, as a personal safety tool

3. Would you recommend the app to others?

- Yes, to people just starting chemsex to prepare (2).
- Yes, useful for people who do not yet know much about the combination of substances.
- Yes, because they can find correct info in there.
- Yes, to keep track of drug usage.
- Yes, to the more careful people like me.
- Yes, because even when you are completely off the map, the app is still very user-friendly. Not too many bells and whistles.
- Yes, I hope it becomes a common tool in the community. It would make parties less risky.

4. General experience| *strenghts*

- Articles
  - Short and concise, very clear
  - Interesting and easily accessible
  - Anyone starting chemsex should be the first to see this
  - Useful information, very practically explained
- Add event
  - Filling in data for an event makes you more aware
- Sufficient privacy
  - You do not need to enter personal email address, common in community to have separate email address for dating profiles

General experience| ***strenghts***

- <u>Notes</u>
  - Very practical to keep track of drug use throughout the event
  - A log for when party gets wilder → kind of logbook from the event
- <u>Drug combinations</u>
  - Easy to use
  - More conscious “I used to take whatever”
  - Scientific basis (resolve disagreements at the party)
- <u>Easy to use</u>
  - Could use the app easily when under the influence

General experience | *points for improvement*

- Articles
  - Add article about:
    - new psychoactive substances
    - slamming (risks and how to avoid them as much as possible)
    - difference between GHB and GBL
  - Add more info on STIs
  - Missing info on safe dosages – general guidelines (per substances: what is a (general) safe range?)
  - Lacking basic information on different substances
  - Add search function

General experience | *points for improvement*

- Buddy
  - Develop further
  - Make it possible to add buddy from contacts
  - Notify Buddy with WhatsApp link? Geo-location?
  - Going somewhere and letting someone I trust know.
  - Notify check-in and/or check-out
  - Message 'I am going to an event and expect to be back at 02h00' and send message 'I am safely home’

- Overview of healthcare services
  - Redirect e-mail addresses
  - Automatic completion of telephone numbers
  - Link addresses to Google Maps or other location service

General experience | *points for improvement*

- <u>Drug combinations</u>
  - Include safety margin doses
  - List in bullets – avoid using paragraphs
  - Simpler wording in layman's terms
  - Adding an extra substance should provide a warning or another safety level (now XTC + cocaine = caution but also XTC + cocaine + speed = caution)
  - Distinguish between GHB and GBL because they are not exactly the same
  - Add substances to the list: cannabis, 2CB

General experience | *points for improvement*

- Emergency
  - Also include 'Call Buddy' here
  - Add standard message 'How do you notify emergency services?' Standard text.
  - Mention to never leave people alone
  - Put 'call emergency services' at the bottom instead of the first option (might scare users)
  - In case of overheating, add ice cubes to cool down

General experience | *points for improvement*

- <u>Notebook</u>
  - Typing difficult under the influence
    - Voice memo?
  - Add purpose: What can the notebook be useful for?
  - Put more central on 'at my party' screen
  - Add terms & conditions: permission for emergency services to access data
  - Mark notes as reminders for the next event
  - Add functionality to delete entrees
  - Add separate section: monitor usage
    - App warns 'You are taking too much too fast’
    - Once product A, then product B: 'Now you're going to take a dangerous combination’
    - Add multiple fill-in boxes: 'What dose?', 'Snorted or injected?', 'What was the impact of this dose?', ...

General experience | *points for improvement*

- <u>‘At my party’</u>
  - Ability to exit event without checking out (access other part of app)
  - Adding a clock 'you've been checked in for so long' or 'total time spent’ → loses sense of time when under influence of drugs
- <u>Calendar</u>
  - Link with personal calendar to plan events
  - Integrate Calendar Budd into personal calendar

General experience | *points for improvement*

- Personal checklist
  - Add extra point: do not take jewellery or any valuable items (or store them safely)
  - Make it visually clear that when you have completed everything you are completely OK to go

- Mood survey
  - Collection of all entered notes and moods so that you can view them chronologically rather than by event
  - Enabling to add moods and notes disconnected from chemsex sessions in the form of a diary

5. Concerns

- Added value
  - How are people going to be encouraged to use the app?
  - People need to be motivated to use the app
  - ‘What’s in it for me?’
  - Add gaming element?

- Privacy
  - Concerned about privacy during event
  - Unnecessary to put info centrally at the top during event? May generate suspicion

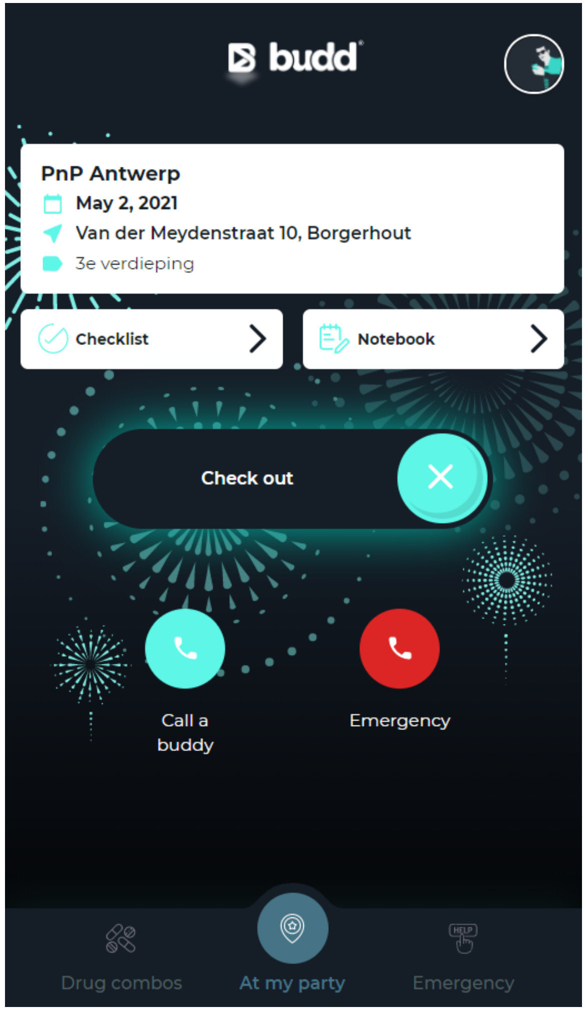

Concerns

- Phone usage during an event
  - Phone not within reach during events (safe location)
  - Sometimes 'not done' to use phone
  - In group of new men: wrong impression to use the app?

- Calendar
  - Unnecessary extra step
  - Maybe useful for people who secretly participate?

- Information module
  - How are users encouraged to go through all the information?
  - Adding a gaming element to test your knowledge

INSTITUTE OF TROPICAL MEDICINE ANTWERP
