# Supplementary material for: Using Intervention Mapping to Develop an mHealth Intervention to Support Men Who Have Sex With Men Engaging in Chemsex (Budd): Development and Usability Study
Source: JMIR Res Protoc. 2022 Dec 21;11(12):e39678. doi: 10.2196/39678 (PMC9813820; doi:10.2196/39678)
Supplement: Multimedia Appendix 7 [file resprot_v11i12e39678_app7.pdf]

Scope 2 Budd: adjustments based on feedback pilot studies

1. **Usability issues**
   - Overview of healthcare services
     - Link addresses to Google Maps (or other location service). This allows users to quickly and easily check whether an organisation is nearby.
   - Articles
     - Each article should have an introduction.
     - Add: search function
   - Safety buddy
     - Adjust entry field mobile phone number: currently unclear that this is a fictitious/sample number: only show ‘04...’
     - Add: possibility to add number from your contacts
   - Settings
     - Make it clear that avatar is clickable
   - My personal checklist
     - Move to settings
     - Make it possible to delete items by sliding
     - 'Add item' is at the top right, in ‘Safety Buddy’ this button is at the bottom. Choose one of the two to ensure continuity within the app.
     - When you want to add an item and go back to the checklist, it is not clear that you need to click the little arrow in the background. Adjust by adding cross on white pop-up screen.
     - Add more clarification: What is the purpose of the personal checklist?
   - Event-specific checklist
     - If you choose either option ('Add personal items' or 'Add items suggested by Budd'), you cannot go back on this choice, only if you delete all items one by one. Customise this by: displaying the user's personal checklist by default, and only displaying the 'items suggested by Budd' button. When the user clicks on it a pop-up (white screen) will appear with items suggested by Budd, the user can then add relevant items one by one.
   - Tips
     - Is currently located too centrally within the app (separate menu). Move this to personal settings or info menu under 'my saved tips'.
   - Add event
     - Make it feasible ('optional') to add time to event.
     - Delete created events simply by swiping.
   - Calendar
     - Also indicate holidays visually.
   - Check-in
     - Visually clarify that you have to use the slider to check-in (the checkmark is confusing).
   - Select your mood
     - Make it visually clear that you can move the dot.

- Drug combination tool
    - Clicking 'Deselect' is too cumbersome to use. Make it possible to deselect in a simpler way (e.g. by adding cross?)
    - It is not sufficiently clear that you can enter multiple combinations, as there is initially only one dropdown menu. Make clearer by e.g. adding a plus sign to the right of the dropdown menu displayed by default.
- Emergency info
    - Red bar 'call emergency services' overlaps text at bottom (not all is readable) put in different place or have it indented with the text.
    - Be more concise and formulate clearer action points. Paragraph is too long (especially when under the influence of substances).
- Notebook
    - Emoticon (survey mood) is displayed too small, the facial expression is difficult to read: display emoticon larger
    - Currently, at least three characters are needed to add a note. Sometimes people work with very short abbreviations so as not to want to write chems in full (e.g. ‘G’), so this should be adjusted to at least one character.
    - Add function: delete/archive note
- Call safety buddy
    - Implement the button 'Call a buddy' at emergency info

2. Adjustments

- Homescreen (becomes ‘dashboard’)
    - Display next event when no event was scheduled on the current day, change to 'upcoming event' instead of 'ongoing event'
- At my party
    - Add a clock/timer showing how long people have already checked in: ‘total time spent’.
    - A way to easily get back to the general section during the event (e.g. make it clear that one is still checked in by displaying the ‘total time spent’ clock in the other sections)
    - Add reminder: ‘don’t forget to check out’ after X number of hours (let user determine this)
    - Display only the event name and date at the top. The location and additional notes do not need to be displayed in this screen (appeared to raise concerns about privacy).
- Drug combinations
    - Simplify wording in layman’s terms
    - Add disclaimer: ‘drugs are never safe’ what we show here is only the expected interaction between substances. No combination is ‘safe’.
    - Add extra warning from 3rd substance added (that it is a greater risk in any case when combining multiple substances)
    - Add cannabis and 2CB to the substances list
- Emergency

    - State that you should never leave persons alone in an emergency situation
- Notes
    - Typing under unfluence is sometimes difficult: enable to save voice messages
    - Make it possible to indicate notes that serve as reminders for the next event
- Personal checklist
    - Add item to ‘let Budd suggest’ list: don’t bring jewellery or valuable items (or make sure you can store them safely)
    - Display in a fun way when people have ticked everything off (sort of ‘well done’?)
    - Reminder on the day of the event: ‘Don’t forget to check your checklist’ (on dashboard)
- Language
    - Replace term ‘drugs’ with ‘chems’ to increase acceptability
    - Tailor choice of words more to the user (now sometimes too academic/difficult)
- Articles
    - Add articles on: new psychoactive substances (NPS) and slamming (risks and how to avoid them as much as possible)
- Add event
    - Delete option ‘host’

3. **New additions**

- Optimisation **Privacy & Cookie Policy**
- **Budd website**, which will include the following:
    - Overview of features
    - Contact information
    - All information articles
    - ‘About the app’ with Frequently Asked Questions:
        - Who developed Budd? How can I start using Budd? What are the benefits of a Budd account? What does the term ‘event’ stand for? How does the app work? Why the choice of the current features? Can I view my data outside the app? Is it safe to rely completely on Budd’s combination tool and chem information? How do I ensure that my data remains private when I leave my phone unattended?
- **Profile management** for people with accounts
- Menu item with **personal statistics** and backup data

- Add **contact form** leading to hello@budd.be so users can ask questions or submit ideas
- Short **experiences/testimonies** from other chemsex participants: can provide a feeling of trust and create a sense of community. Kind of 'chemsex stories' where user can click on. Should be possible to add new testimonials. Could attract people to go to the app again 'there's a new story'. → categorised under 'Chemsex stories' in the information module. Same structure as an article and under each testimonial comes: 'Do you want to share your story? Send it to...'.
- Add an **article 'Welcome to Budd!'** where we answer some frequently asked questions to new users, such as: How can I use Budd?, How does Budd work?, What are the benefits of a Budd account?, Is it safe to fully rely on Budd's combination tool and chem information?, Why the choice of the current features?, and Who developed Budd?.
- **Knowledge quiz** (will be about the information in the articles, emergency info and drug combinations). Users get a score and can retake this quiz to achieve an 'expert profile'. This is to motivate users to go through the information offered in the app.
- **General info on most used chemsex drugs**. For each substance: What is it (description of substance)? What is a low, medium and strong dose (per form of administration)? What effects can be expected? How long do the effects last? Add 'Chems' button in the information menu.
- The profile should be given a more prominent place; **'my personal dashboard'**: possibility to view all collected data in summary form in an overview (averages, evolution of moods and participation in events, all journal/diary notes). Overview of data and summary. Dashboard should contain:
    - Next event
    - Shortcut(s) to events
    - Joural/diary based on notes per event and moods
    - Entry in journal (=note): option to enter moods or a journal entry (text only)
    - Notifications: New articles? Quizzes not yet filled in?
    - My personal checklist

- **Preparation tool** (next page)

Preparation tool proposal

The user is asked three questions per theme. Next to each question is an info-button that can be clicked if one wants more information about the relevant preparation strategy. The user can fill in these questions without obligation in preparation for the chemsex event. From the questions filled in, a question will be randomly selected after the event. The question and corresponding answer will be shown to the user, after which he can indicate whether he succeeded in his preparation, with the possibility of reflecting on it.

ⓘ = extra info per question

Preparation tool

Use this tool to prepare yourself for your participation in [name event].

1. Practical

How much time did I plan after the event to recover?
ⓘ In order to counteract the negative effects of chems as much as possible, you can choose to strategically plan parties in your personal agenda. Broadly speaking: try to take it easy the days before a party, and plan time to recover the days after. Do you have important plans shortly after the party? Or have you just had a very exhausting couple of days? Then you'd be better off cancelling this party and looking for one at a better time.

When do I intend to go home? *You can set an alarm on your phone to alert you.
ⓘ When you have taken chems you have less sense of time. Because of this you can go on for hours or days without fully realising it. Try to determine in advance at what time you would prefer to go home. You can also take a limited amount of chems with you, as well as a budget so that it is more difficult to make extra purchases at the party. In addition, try to avoid scrolling on dating apps again on the way home. This way you avoid being tempted to go to a new party. You can also involve a friend to come home with you, or notify a person who is not at the party that you will be coming home at a certain time.

How will I get home safely after the event?
ⓘ Avoid driving under the influence of chems. The following options will ensure that you arrive safely at home (consider these beforehand):

- Use public transport: No, it’s not glamorous, but taking the bus, metro or train home is cheaper than a cab and safer than driving yourself.
- Pick a designated driver: Designate a bob at the beginning of the party and stick to it. Just make sure that friend hasn’t drunk too much! Don’t ride with anyone who’s intoxicated, even if they say they’re fine.

- Take a taxi: When you are far from home and are under the influence of drugs, it is advisable to arrange a taxi. It may seem expensive, but a safe trip home is more than worth it.
- Spend the night: If there are no other options to get home safely, you can ask if you can crash on the couch.

2. Chems

Which chems do I certainly not want to take during the event?

ⓘ Personal limits can blur under the influence of chems and the intense feelings of sexual excitement during a chemsex party. It can therefore be useful to clearly name and identify your personal limits beforehand. When these are clearly defined, you are more likely to stick to them.

Do I know enough about the risks and harm reduction strategies of the chems I want to take?

Am I going to bring my own chems and material?

ⓘ Bring everything you will need during the party so you don't have to rely on others. Make sure to have easy access to condoms and lube. Bring your own drinking straw, sniff tube and needles (if applicable). Make sure you can easily identify these items as yours. Don't share them with others. It may also be a good idea to bring your own chems so that you know the quality and are not faced with unpleasant surprises. Also think of bringing a phone charger or charge pack. If you take HIV medication or PrEP, make sure you have a supply on you.

3. Sex

Have I set an alarm for my medication intake? (if applicable)

ⓘ When taking HIV medicines it is important to take them as close as possible to the same time each day. Missing doses can reduce their usefulness and increase the possibility of developing drug resistance, which makes certain HIV drugs lose their effectiveness. PrEP is also best taken around the same time each day to be the most effective. Chems can influence your perception of time. You can solve this problem by setting an alarm on your smartphone to remind you to take your medicines.

What am I prepared to do sexually?

ⓘ Personal limits can blur under the influence of drugs and the intense feelings of sexual excitement during a chemsex party. It can therefore be useful to clearly name and identify your personal limits beforehand. When these are clearly defined, you are more likely to stick to them.

Did I discuss sexual preferences with my sexual partners in advance?
